# Bioinspired wearable polymer microneedle patches: pioneering diabetic wound therapy for the horizon

**DOI:** 10.1039/d5ra02557e

**Published:** 2025-09-08

**Authors:** Shana Zhang, Meng Wei, Chengxin Luan, Bingbing Gao

**Affiliations:** a Department of Clinical Laboratory, Capital Medical University Electric Teaching Hospital, Beijing Electric Power Hospital Beijing 100073 China zhangshana@gt.cn; b School of Pharmaceutical Sciences, Nanjing Tech University Nanjing 211816 China gaobb@njtech.edu.cn; c Department of Hematology, The Fourth Division Hospital of Xinjiang Production and Construction Corps Yining People's Republic of China

## Abstract

Diabetic wounds present persistent challenges due to impaired healing, recurrent infection, oxidative stress, and dysregulated glucose metabolism. Bioinspired polymeric microneedle (MN) patches have emerged as multifunctional platforms capable of penetrating the stratum corneum to deliver therapeutics directly into the dermis, enabling glucose regulation, antimicrobial action, reactive oxygen species (ROS) modulation, and proangiogenic stimulation. Recent experimental evidence has demonstrated that the integration of glucose oxidase-loaded porous metal–organic frameworks, photothermal nanomaterials, and antioxidant hydrogels within dissolvable MNs achieves synergistic bactericidal effects, accelerates collagen deposition, and enhances neovascularization in diabetic wound models. Stimuli-responsive designs facilitate precise and sustained drug release while reducing off-target effects. Structural innovations, including hollow, multilayer, and bioinspired morphologies, improve mechanical compliance, drug loading, and patient comfort. Despite promising *in vivo* healing outcomes and improved microenvironment regulation, large-scale manufacturing, long-term stability, and clinical translation remain key challenges. This review highlights advances in MN materials, structures, and mechanisms, providing insights for the development of next-generation intelligent wound therapies.

## Abstract 2

Diabetic wounds remain difficult to heal due to impaired repair, recurrent infection, oxidative stress, and disrupted glucose regulation.

## Graphical abstract

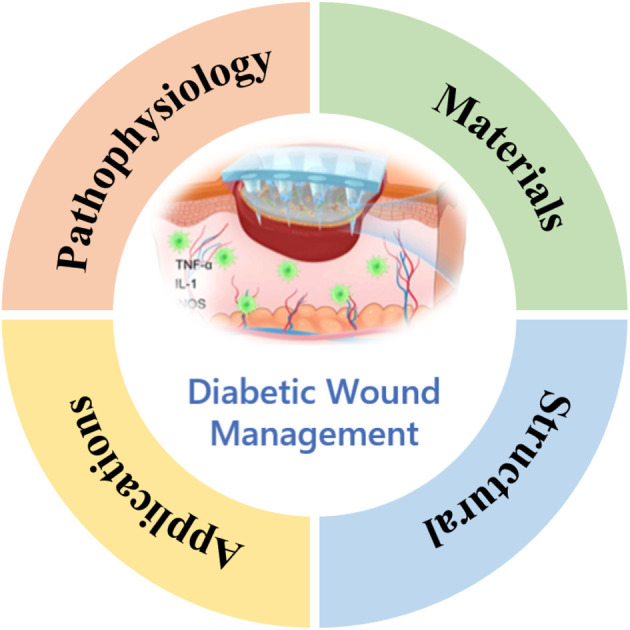

## 1. Introduction

Chronic wounds are a serious and escalating healthcare problem, affecting millions of people worldwide and placing a substantial burden on healthcare systems.^1–3^ Among these, diabetic wounds are particularly problematic because they exhibit slow healing, high recurrence rates, and an elevated risk of severe complications, including infection, gangrene, and amputation.^4–7^ These wounds arise from a combination of systemic and local pathological factors, such as persistent hyperglycemia, impaired immune function, vascular insufficiency, and neuropathy, all of which disrupt the orderly progression of wound healing stages. The wound microenvironment in diabetic patients is typically characterized by prolonged inflammation, excessive protease activity, elevated reactive oxygen species (ROS), and recurrent bacterial colonization. These abnormalities not only delay angiogenesis and collagen deposition but also hinder tissue remodeling,^8–11^ making diabetic wounds a persistent clinical challenge. Conventional treatments, including debridement, topical antimicrobials, systemic antibiotics, and growth factor dressings, offer only partial benefits and often fail to address the multifactorial nature of these wounds. Furthermore, poor drug penetration into infected tissues, rapid degradation of bioactive molecules, systemic side effects, and the need for frequent hospital visits reduce therapeutic efficacy and patient adherence, creating an urgent need for advanced, integrated wound care strategies that can intervene at multiple biological levels.^12^

In recent years, microneedle (MN)-based transdermal delivery systems have gained significant attention as a minimally invasive and efficient alternative to traditional therapies.^13–16^ By creating microchannels in the stratum corneum, MNs allow rapid and localized delivery of therapeutic agents into the dermis without reaching pain receptors or blood vessels, thereby reducing discomfort, anxiety, and infection risk.^17–20^ This approach combines the advantages of hypodermic injections and topical administration while overcoming many of their limitations. Advances in materials science, microfabrication, and drug formulation have enabled MN platforms to incorporate multiple therapeutic functions, including glucose regulation, antimicrobial delivery, ROS scavenging, and pro-angiogenic stimulation—capabilities that directly address the complex pathology of diabetic wounds.^13,21–23^ For example, multifunctional MNs can carry glucose oxidase to lower local glucose concentrations, release antimicrobial agents to control infection, and deliver growth factors to promote angiogenesis and tissue regeneration. Some designs even integrate nanoparticles, hydrogels, or responsive polymers to enhance stability and prolong drug release. However, most existing MN designs focus on single therapeutic targets, which limits their ability to modulate the wound environment comprehensively. Material selection also requires balancing mechanical strength, biocompatibility, biodegradability, and drug-loading efficiency, while large-scale manufacturing, long-term stability, and cost-effectiveness remain barriers to clinical translation. Moreover, the integration of real-time sensing and stimuli-responsive release—essential for personalized and adaptive therapy—remains at an early stage, and its potential in wound management is far from fully realized.

**Fig. 1:**
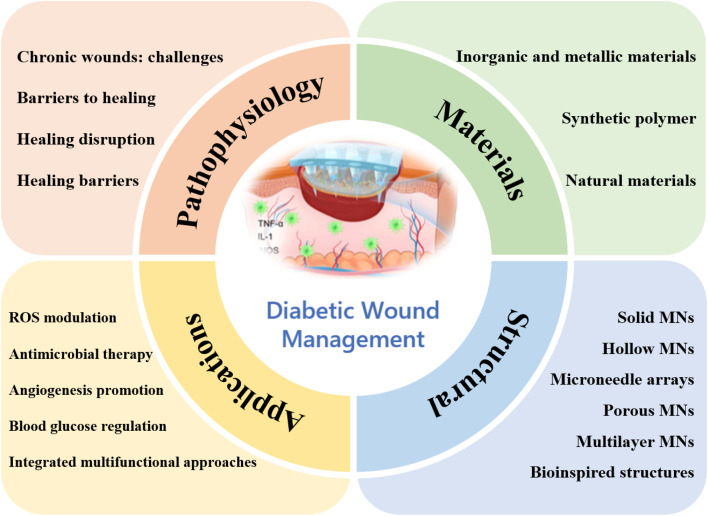
Multifunctional microneedle patch for diabetic wound therapy.

Bio-inspired polymeric MN patches offer a versatile and promising platform to overcome these challenges by combining multifunctional therapeutic strategies with adaptive release mechanisms.^24–26^ Drawing inspiration from natural structures, these systems can optimize mechanical compliance, fluid management, and drug distribution, while integrating responsive components that react to biochemical cues such as glucose concentration, pH shifts, or ROS levels in the wound microenvironment. For instance, glucose oxidase-loaded porous metal–organic frameworks embedded in dissolvable polymer MNs can not only deplete excess glucose but also generate bactericidal hydrogen peroxide and co-deliver antimicrobial compounds, effectively addressing both hyperglycemia and infection while supporting tissue repair. Other approaches integrate angiogenic peptides, anti-inflammatory agents, or oxygen-generating materials to further accelerate healing. Such designs improve therapeutic efficacy, reduce treatment frequency, and enhance patient comfort and compliance. In addition, the adaptability of bio-inspired MNs allows for customized treatment regimens based on wound type, size, and stage of healing, paving the way for truly personalized wound care. This review provides a comprehensive overview of the materials, structural designs, mechanisms of action, and therapeutic performance of bio-inspired polymeric MN patches for diabetic wound therapy (Fig. 1).^27–30^ We critically examine their benefits, limitations, and translational prospects, with the aim of guiding the development of next-generation MN platforms that integrate multifunctionality, precision delivery, and patient-centered usability. By bridging the gap between laboratory innovation and clinical application, this work seeks to advance the field toward effective, convenient, and accessible solutions for the management of diabetic wounds, ultimately improving patient outcomes and reducing the societal impact of chronic wound care.^31–33^

## 2. Pathophysiology of chronic and diabetic wounds

### 2.1 Clinical and pathophysiological challenges of chronic wounds

Chronic wounds represent significant challenges for the medical system, as they are prevalent conditions associated with high morbidity rates. These wounds are characterized primarily by prolonged healing periods, which can last for months or even years, making them persistent issues for patients and healthcare providers alike. The complexity of chronic wounds arises from their multifactorial nature;^34–37^ they can be caused by a variety of factors that interact in intricate ways. Frequently, these wounds involve complications such as infections, which can further impede the healing process.^38–40^ The presence of infections not only prolongs the duration of the wound but also complicates treatment strategies, as they often require aggressive intervention to eradicate pathogenic organisms. In addition to infections, circulatory disorders play crucial roles in the development and persistence of chronic wounds.^41,42^ Conditions such as peripheral artery disease can reduce blood flow to the affected area, depriving the tissues of the essential nutrients and oxygen needed for effective healing. Similarly, venous insufficiency can lead to fluid accumulation and tissue swelling, exacerbating the wound condition. Necrotic tissues can complicate the healing process, as dead or dying tissue serves as a barrier to healing and provides a breeding ground for bacteria. The presence of necrosis not only hinders the body's natural healing mechanisms but also necessitates additional surgical interventions or advanced wound care techniques to remove the devitalized tissue. The interplay of these diverse factors makes chronic wounds a complex clinical challenge, requiring comprehensive and multifaceted approaches for treatment.

### 2.2 Pathophysiological barriers in diabetic wound healing

The difficulty in healing diabetic wounds can be attributed to several interrelated factors. First, the chronic hyperglycemic state characteristic of diabetes leads to metabolic imbalances that impair cellular function and disrupt the normal phases of wound healing.^43,44^ Elevated blood sugar levels can compromise the activity of immune cells, reducing the body's ability to fight infections and respond effectively to tissue damage. Diabetic patients frequently experience peripheral neuropathy, which diminishes sensation in the extremities. This loss of feeling can result in unnoticed injuries, allowing wounds to develop and worsen without prompt attention. Combined with poor circulation, these factors create a vicious cycle in which minor injuries can escalate into significant wounds that are slow to heal. Diabetic wounds are characterized by a high recurrence rate. Even after successful treatment, the underlying conditions that contribute to wound formation remain, making patients susceptible to new wounds.^45,46^ This recurrence not only poses ongoing health risks but also places a substantial emotional and financial burden on patients and healthcare systems. Diabetic wounds exemplify the challenges of chronic wounds, as they not only involve complex healing dynamics but also significantly affect the quality of life of those living with diabetes. Addressing these wounds requires a comprehensive understanding of the underlying factors and tailored treatment approaches to improve healing outcomes and reduce the likelihood of recurrence.

### 2.3 Disruptions in the healing stages of diabetic patients

During the normal process of wound healing, cutaneous regeneration typically occurs in a systematic manner and involves four distinct but interrelated stages: hemostasis, inflammation, cell proliferation, and matrix remodeling. Each of these phases plays a crucial role in ensuring effective repair and restoration of the integrity of the skin. Hemostasis is the initial response to injury, where the body's primary goal is to prevent blood loss. When a wound occurs, blood vessels constrict, and platelets aggregate at the site to form a clot. This clot not only serves as a physical barrier to pathogens but also releases signaling molecules that initiate the healing process.^47^ The clotting cascade activates various proteins that help stabilize the wound area and lay the groundwork for subsequent healing phases. Following hemostasis, the inflammatory phase begins, typically lasting for several days. During this stage, the body sends immune cells, such as neutrophils and macrophages, to the wound site. These cells play a vital role in cleaning wounds by removing debris, bacteria, and dead tissue. Inflammation is characterized by redness, heat, swelling, and pain, which are signs of increased blood flow and immune activity. This phase is essential for preventing infection and setting the stage for tissue regeneration. The third phase, cell proliferation, focuses on tissue repair and regeneration. During this stage, fibroblasts and endothelial cells migrate to the wound area. Fibroblasts are responsible for synthesizing collagen and other extracellular matrix components that provide structural support to new tissues. Moreover, endothelial cells help form new blood vessels, a process known as angiogenesis, which ensures an adequate blood supply to the healing tissue. Additionally, epithelial cells migrate across the wound surface to cover it, facilitating the reestablishment of the skin barrier. The wound enters the matrix remodeling phase, which can last for months or even years. During this stage, the initially formed collagen matrix is remodeled and strengthened. Collagen fibers reorganize, cross-link, and mature, leading to increased tensile strength of the repaired tissue. This phase is crucial for restoring the structural integrity and function of the skin, ultimately resulting in the formation of a scar. The remodeling process is dynamic, with continuous turnover and adaptation of the collagen matrix, allowing the tissue to respond to mechanical stresses and maintain homeostasis.

### 2.4 Factors contributing to delayed healing and recurrence

The process of normal wound healing is complex and highly coordinated, involving a series of well-defined stages that together facilitate effective cutaneous regeneration.^48,49^ Each phase is essential for ensuring that the wound heals properly and efficiently, highlighting the body's remarkable ability to repair itself in response to injury. However, diabetic wounds do not adhere to the typical healing sequence observed in healthy individuals. Instead, their healing process is significantly disrupted because of the persistent hyperglycemic state associated with diabetes. Elevated blood sugar levels create an environment that not only promotes the growth of bacteria but also leads to a variety of complications that hinder effective wound healing. In a hyperglycemic environment, the increased availability of glucose can fuel bacterial proliferation, resulting in increased infection rates. This overgrowth of bacteria contributes to a vicious cycle in which infections further complicate the wound and delay healing. The presence of pathogens triggers an exaggerated immune response, leading to an imbalance in the wound microenvironment. This imbalance is characterized by the overproduction of proinflammatory cytokines, which are signaling molecules that regulate inflammation and immune responses. The excessive release of these proinflammatory cytokines can have detrimental effects on the healing process. Instead of facilitating repair, they can exacerbate inflammation, prolonging the inflammatory phase far beyond what is considered normal. In addition, elevated levels of proteases—enzymes that breakdown proteins—can lead to the degradation of important structural components within the wound. This not only impairs tissue integrity but also contributes to the overall failure of the healing process.^50,51^ Compounding these issues involves the inhibition of growth factors, which are crucial for various aspects of wound healing, including cell migration, proliferation, and the formation of new blood vessels. In diabetic wounds, the production of these vital growth factors is often significantly reduced, further stalling the transition from the inflammatory phase to the proliferative phase of healing. Without adequate levels of growth factors, the wound fails to progress effectively, leading to chronic nonhealing ulcers. The result of this complex pathology is a prolonged and exacerbated inflammatory phase, where the wound remains in a state of persistent inflammation rather than progressing toward healing. This not only increases the risk of complications such as further infections and necrosis but also contributes to the high recurrence rates associated with diabetic wounds. As a result, managing diabetic wounds requires a multifaceted approach that addresses both the underlying metabolic issues and the specific challenges presented by the impaired healing process.

## 3. Microneedle materials for diabetic wound therapy

### 3.1 Inorganic and metallic materials

#### 3.1.1 Silicon

The application of silicon materials in microneedle systems has garnered significant attention because of their exceptional mechanical properties, structural precision, and compatibility with microfabrication techniques, particularly demonstrating notable advantages in wound treatment related to diabetes and continuous glucose monitoring. A transdermal sensing platform based on high-density silicon microneedle arrays can stably penetrate the skin barrier to extract interstitial fluid for highly sensitive electrochemical detection. In animal experiments, these results are highly consistent with those of traditional blood glucose measurement methods, suggesting a noninvasive, continuous blood glucose monitoring alternative.^52^ Silicon microneedles also possess excellent electrical insulation properties and micro/nanopatterning capabilities, facilitating the integration of sensing electrodes and functional coatings on their surfaces to enable simultaneous detection of multiple physiological parameters, providing real-time monitoring and personalized treatment feedback for complex diabetic wound environments.^53^ Although silicon itself lacks biodegradability and has high manufacturing costs, limiting its application in large-scale clinical deployment, recent advancements in low-cost fabrication methods under noncleanroom conditions, combined with strategies involving biodegradable hydrogels, have significantly improved its manufacturability and biocompatibility, making it practically feasible for structural support, controlled release, and multimodal drug delivery.^54^ Earlier studies have also demonstrated the safety and efficacy of MN platforms in transdermal drug delivery, providing a crucial foundation for the rapid development of functional integrated MN systems today.^55^

#### 3.1.2 Stainless steel

**Fig. 2:**
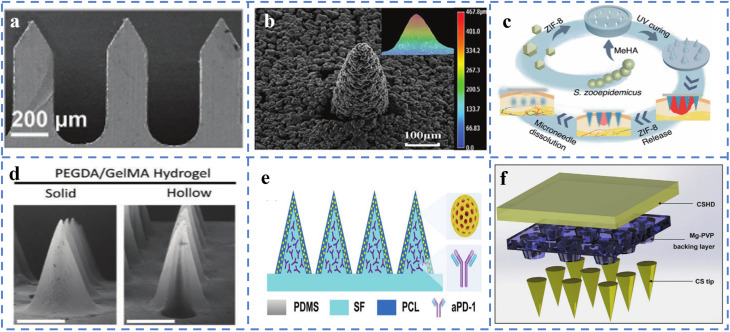
Materials of microneedles. (a) Use of stainless steel.^56^ Reprinted with permission. Copyright 2024 Wiley-VCH GmbH. (b) Enlarged scanning electron microscope images of porous titanium microneedles.^62^ Reprinted with permission. Copyright 2025 Wiley-VCH GmbH. (c) The use of hyaluronic acid.^65^ Reprinted with permission. Copyright 2021 Wiley-VCH GmbH. (d) Synthesis of microneedles with two structures from polyethylene glycol diacrylate.^54^ Reprinted with permission. Copyright 2023 Wiley-VCH GmbH. (e) Use of silk fibroin.^79^ Reprinted with permission. Copyright 2022 Wiley-VCH GmbH. (f) Use of chitosan.^83^ Reprinted with permission. Copyright 2022 Wiley-VCH GmbH.

Owing to their excellent mechanical strength, electrical conductivity, and structural stability, stainless steel materials show great potential for use in microneedle systems, especially in the treatment of chronic wounds related to diabetes (Fig. 2a). Compared with flexible materials such as polymers or hydrogels, stainless steel MNs have stronger skin penetration capabilities and long-term stability, making them suitable for multiple functions, such as drug delivery, electrical stimulation, and biological signal monitoring. In an integrated intelligent wound treatment system, stainless steel MNs are designed as conductive scaffolds that synergistically form a dual-layer structure with soluble drug MNs. Their conductivity is enhanced through coatings of carbon nanotubes and silver, enabling the sustained release of antimicrobial drugs while simultaneously monitoring key wound metabolites such as hydrogen peroxide and uric acid in real time. Additionally, triboelectric nanogenerators provide electrical stimulation to accelerate cell proliferation and tissue repair, effectively promoting wound healing in diabetic patients.^56^ Additionally, in drug delivery research, computational modeling and Franz diffusion cell experiments revealed that the insertion method and retention time of stainless steel MNs significantly influence drug diffusion efficiency, suggesting that precise control of MN pressure conditions is necessary in clinical applications to ensure effective drug release and tissue response.^57^ Although stainless steel itself is nonbiodegradable and poses potential risks of metal ion leaching, researchers have successfully avoided safety hazards associated with long-term biological contact through functional surface modification and structural interlayer isolation strategies, making it more suitable as an integrated diagnostic and therapeutic module rather than a direct drug-carrying entity.^58^ The role of stainless steel MNs in diabetic wound treatment is evolving from traditional mechanical puncture tools to key components of intelligent diagnostic and therapeutic platforms, providing more efficient, precise, and controllable comprehensive intervention methods for chronic wounds.

#### 3.1.3 Titanium

Titanium materials have increasingly emerged as highly promising metallic materials for microneedle structures in recent years because of their exceptional biocompatibility, superior mechanical properties, corrosion resistance, and adaptability in micro- and nanoprocessing (Fig. 2b). They have demonstrated significant advantages, particularly in the treatment of chronic wounds associated with diabetes. The high strength-to-weight ratio of titanium gives it ideal skin penetration capabilities, whereas its inert surface reduces the risk of inflammatory reactions and tissue rejection, making it highly suitable for therapeutic microneedle platforms requiring frequent or prolonged skin contact. In terms of MN manufacturing processes, coating silicon-based MNs with titanium can significantly increase their surface smoothness and structural stability, thereby improving their skin insertion performance and mechanical reliability. For example, in microneedle structures prepared *via* dry etching, coating with titanium not only improves the surface roughness of the needle body but also results in lower displacement rates and higher fracture resistance in mechanical testing, ensuring the safety and durability of the microneedles during application.^59^ In terms of therapeutic functionality, titanium materials possess excellent multifunctional modification potential, enabling their use as acoustic sensitizers, photodynamic agents, or electrode materials in the construction of controlled-release and dynamic-response systems. A titanium oxide (TiO_2−*x*_)–glucose oxidase coloaded ultrasonic-responsive microneedle system successfully achieved local release of carbon monoxide gas in diabetic infected wounds, effectively accelerating wound healing through synergistic antibacterial and tissue repair effects. This system leverages the ultrasonic-responsive properties of titanium-based materials to trigger the decomposition of CO prodrugs under ultrasonic stimulation, effectively inhibiting *Staphylococcus aureus* and methicillin-resistant *Staphylococcus aureus* (MRSA) in high-glucose, high-oxidative stress microenvironments while promoting fibroblast migration and proliferation.^60^ Additionally, titanium materials can be used to construct responsive diagnostic and therapeutic platforms that release active molecules in the acidic microenvironment of infected wounds and, in conjunction with oxidative stress regulatory mechanisms, penetrate and eliminate bacterial biofilms. For example, near-infrared fluorescent probes based on titanium undergo structural transformations in acidic environments, enabling infection detection while synergistically inducing reactive oxygen species production and triggering carbon monoxide release, demonstrating the highly integrated functionality of titanium materials in ‘diagnosis–treatment’-integrated microneedle systems.^61^ Additionally, the conductivity of titanium materials enables the development of electrically stimulated microneedles, which enhance the regenerative efficiency of wound microenvironments, synergistically enhancing drug release and tissue repair mechanisms, making them a key component in the construction of electroactive microneedle systems.^62^ Titanium materials not only provide structural support and mechanical assurance but also lay the functional foundation for achieving multimodal, intelligent treatment with MNs in diabetic wounds, representing an important direction for the future development of high-performance therapeutic MNs.

### 3.2 Polymer materials

#### 3.2.1 Hyaluronic acid

Hyaluronic acid (HA), a natural anionic polysaccharide, has become one of the most promising biomaterials for constructing microneedle systems because of its excellent biocompatibility, biodegradability, moisturizing properties, and easy chemical modification of its molecular structure. It is particularly suitable for complex treatment scenarios such as chronic diabetic wounds, which involve multiple factors (Fig. 2c). Among various types of HA-based MN platforms, by subjecting HAs to methylation, photopolymerization, or composite processing with functional nanomaterials, customizable hydrogel MN structures can be formed that combine mechanical penetration performance, drug delivery capability, and multifunctional therapeutic effects, fully addressing the comprehensive treatment requirements of diabetic wounds, including antibacterial, antioxidant, and proangiogenic functions.^63^ Studies have shown that loading dopamine-functionalized nanoparticles (PDA NPs) and iron-based mesenchymal stem cell-derived nanovesicles (Fe-MSC-NVs) rich in therapeutic cytokines into the shell and core layers of HA MNs, respectively, enables responsive degradation and sustained release in diabetic wound environments. This not only significantly inhibits chronic inflammatory responses induced by reactive oxygen species but also promotes the migration, proliferation, and angiogenesis of human umbilical vein endothelial cells while inducing M2 macrophage polarization, thereby creating a favorable tissue regeneration microenvironment.^64^ Additionally, HA MNs can be loaded with metal–organic framework (MOF) structures, such as Zn–MOF, to confer excellent antibacterial properties and stable drug release characteristics. In the context of diabetic wound infections, Zn^2+^ release can disrupt bacterial capsules and alleviate oxidative stress. Photocrosslinked MeHA-based MNs further ensure stable mechanical properties and degradability, enabling the entire MN system to achieve efficient epithelial regeneration and neovascularization without causing secondary damage.^65^ In addition to traditional drug-loaded MNs, HA is also suitable for constructing multimodal intelligent response systems. For example, by leveraging the photothermal conversion capability of MXenes, they can be coencapsulated with IL-17 monoclonal antibodies within HA MNs. Under near-infrared light irradiation, the microneedles rapidly heat up and dissolve, enabling deep delivery of biopharmaceuticals and precise suppression of local immune inflammation. Although this strategy is primarily used for psoriasis treatment, it has high transferability in addressing focal chronic inflammation in diabetic wounds.^66^

#### 3.2.2 Polylactic acid

Polylactic acid (PLA) is a biodegradable polymer derived from renewable resources. Owing to its excellent biocompatibility, mechanical strength, and processability, it has garnered significant attention in recent years, particularly in microneedle systems, especially those used in microneedle platforms for the treatment of chronic wounds associated with diabetes. PLA possesses high rigidity and moderate brittleness, enabling it to maintain skin penetration capability while achieving controlled biodegradation behavior through adjustment of its molecular weight, crystallinity, and processing parameters. This makes it suitable for constructing microneedle carriers with stable structures, strong drug-loading capacity, and controlled release characteristics. In MN systems that integrate sensing and therapeutic functions, polymer-based MNs offer greater biocompatibility and manufacturing flexibility than traditional metal or inorganic materials do. Research shows that acrylic solid microneedles prepared *via* 3D printing technology can be further combined with conductive polymer coatings to construct all-polymer conductive microneedle arrays, enabling microneedle electrode functionality without relying on metal layers. This approach retains the excellent mechanical properties and tissue compatibility of PLA-based substrates while enabling continuous monitoring of biomarkers, providing a platform foundation for real-time inflammation monitoring and healing assessment in diabetic wounds.^67^ Additionally, owing to their injectable, photopolymerizable, and layer-by-layer manufacturing characteristics, poly(lactic acid)-based materials are particularly suitable for the development of customized wearable trauma diagnostic devices for small-scale production. Moreover, the potential of microneedle-sensing platforms in the management of chronic diseases such as diabetes continues to expand, particularly in the noninvasive, high-throughput, and multichannel detection of biochemical information such as glucose, lactate, and pH in interstitial fluid. This review highlights that microneedle systems constructed using biodegradable polymers such as PLA have made significant progress in terms of material engineering, sensor integration, and biosignal processing. Their flexibility and miniaturization capabilities make them an important component of “microneedle lab-on-a-chip: technology”, which is particularly suitable for real-time acquisition and feedback regulation of complex multicomponent signals in diabetic wound environments.^53^ Therefore, functional MN platforms based on polylactic acid not only combine structural support, sensing capabilities, and biocompatibility but also demonstrate significant application potential in precise treatment, continuous monitoring, and intelligent feedback for chronic diabetic wounds. In the future, through deep integration with conductive materials, bioactive molecules, and visualization technologies, higher levels of personalized wound management can be achieved.

#### 3.2.3 Polyethylene glycol diacrylate

Polyethylene glycol diacrylate (PEGDA) is a photopolymerizable synthetic polymer hydrogel material that has been widely used in the construction of MN systems because of its excellent biocompatibility, tunable physical properties, good moldability, and low immunogenicity (Fig. 2d). This technique has shown significant application potential, particularly in the treatment of chronic wounds associated with diabetes. PEGDA can be rapidly cured under low-energy conditions *via* photopolymerization, making it suitable for high-resolution structural construction, particularly in the preparation of complex microstructures such as hollow or biomimetic array microneedles. On this basis, researchers have developed a microneedle array platform using a composite material of PEGDA and gelatin methacrylate (GelMA), employing low-cost 3D printing and thermal shrinkage mold replication processes, enabling the scalable production of hollow and solid microneedles of various sizes without relying on cleanroom conditions. This provides diabetic patients with an affordable, customisable therapeutic tool that combines drug injection and sustained-release capabilities.^54^ To achieve further drug release visualization and monitoring functionality, another study utilized PEGDA to replicate templates with antivescent structures, constructing anticoalescent microneedle arrays (IOMNs) with interconnected porous structures. This structure not only enhances the drug loading capacity but also results in blueshifting structural color changes due to refractive index alterations during drug release, enabling real-time visual monitoring of the drug release process. This provides intelligent therapeutic feedback for chronic wounds, particularly diabetic wounds.^68^ Research has further incorporated biofunctional materials to endow the microneedle system with antimicrobial and antioxidant therapeutic functions. For example, PEGDA was combined with melanin-loaded hydrogels to construct microneedle patches with antioxidant and visual sensing capabilities synergistically integrated with gelatin-based antibiotic nanopore materials to achieve ROS clearance and drug delivery functions for bacterially infected wounds, significantly enhancing the repair efficiency of diabetic wounds.^69^ Additionally, a microneedle material system copolymerized from PEGDA and GelMA has been used to deliver modified adipose-derived stem cell exosomes (ADSC-EVs), constructing a separable microneedle patch platform. The soluble hyaluronic acid substrate rapidly detaches under the action of exudate, enabling sustained release of active factors by the needle body within the wound, reversing cellular senescence in diabetic wounds, improving the PTEN/PI3K/AKT signaling pathway, and enhancing tissue regenerative capacity.^70^

### 3.3 Natural materials

#### 3.3.1 Trehalose

Trehalose, a natural nonreducing disaccharide, demonstrates significant protein stabilization and drug protection capabilities in soluble MNs, making it particularly suitable for scenarios requiring high drug activity and delivery efficiency in the treatment of chronic wounds associated with diabetes. Its glass transition properties effectively maintain the conformation integrity of biomolecules such as proteins and virus-like particles (VLPs), enabling vaccines or therapeutic agents to remain stable at room temperature without inactivation. Additionally, it facilitates the sustained release of these agents in skin or mucosal tissues, inducing prolonged immune responses and tissue repair.^71^ By combining with biomimetic structures, trehalose forms an outer shell layer in egg-shaped MNs, simulating a protein-stabilizing environment, effectively protecting the activity of liraglutide during MN preparation and achieving complete delivery. Its blood sugar-lowering effect in a diabetes model is comparable to that of subcutaneous injection.^72^ Additionally, by integrating a simple applicator capable of precisely releasing elastic strain energy, the efficiency of MN insertion and drug release rates are significantly increased, providing a controllable platform for the efficacy of trehalose stabilizers.^73^ Furthermore, the concept of soluble MNs as ‘*in situ* chemical reaction chambers’ has expanded the potential of trehalose in regulating the local microenvironment, enabling enzymatic reactions, or antioxidant therapy, particularly in diabetic wounds rich in harmful metabolites and chronic inflammation.^74^ The application of trehalose in MN systems not only enhances the stability and therapeutic efficacy of biopharmaceuticals but also offers highly promising prospects for personalized and accessible treatment of diabetic wounds.^75^

#### 3.3.2 Silk fibroin

Silk fibroin exhibits a unique combination of biocompatibility, mechanical robustness, and controlled degradability, making it an ideal candidate for constructing multifunctional microneedle (MN) platforms tailored for chronic diabetic wound care. Through rational structural design and molecular functionalization, silk-based MNs have been demonstrated to be capable of staged drug release, immunomodulation, and microenvironment-responsive therapy. For example, a silk fibroin methacryloyl MN system incorporated Prussian blue nanozymes and VEGF in needle tips to address oxidative stress and promote angiogenesis, whereas polymyxin-loaded bases provided antibacterial protection, collectively accelerating diabetic wound closure.^76^ In a separate approach, a nanofiber-based core–shell silk fibroin MN enabled sequential immunosuppression and regeneration by releasing a BRD9 degrader followed by a Hedgehog pathway agonist.^77^ Furthermore, integration with stimuli-responsive hydrogels and flexible electronics has expanded MN functionality beyond drug delivery—for example, a temperature-responsive silk MN dressing combined with photonic crystals, microfluidics, and wireless sensors for real-time inflammation monitoring and therapeutic feedback.^78^ The versatility of silk fibroin is also evidenced by its successful adaptation to wearable platforms for programmed hydrogen therapy and immunotherapy^79^ and its use in spatially resolved neuroactive drug delivery.^80^ Even in plant systems, silk MNs enable precise phytohormone delivery, highlighting the material's cross-domain potential.^81^ Collectively, these studies underscore the growing utility of silk fibroin as a smart, sustainable matrix for MN-based interventions targeting the multifactorial pathology of diabetic wounds.

#### 3.3.3 Chitosan

Chitosan (CS), a natural cationic polysaccharide, has been widely used in the development of functional microneedle systems because of its excellent biocompatibility, biodegradability, and antimicrobial properties, particularly in addressing the complex inflammatory and infectious microenvironments associated with chronic diabetic wounds (Fig. 2f). In one study, researchers developed a dual-layer microneedle patch in which the needle tips were composed of a chitosan–gelatin composite loaded with epidermal growth factor (rh-EGF), while the basal layer released tetracycline (TH). Through gelatinase-responsive release, rh-EGF is released in a time-controlled manner, effectively inhibiting inflammation, promoting angiogenesis, and stimulating collagen production, thereby accelerating wound healing.^82^ Another design utilized CS to construct a synergistic removable microneedle system, combining soluble polyvinylpyrrolidone (PVP) with a magnesium layer to release ginsenosides (PNS) to promote immune regulation and angiogenesis and alleviate chronic inflammation through local pH neutralization, achieving dynamic regulation throughout the entire healing process.^83^ Additionally, CS has unique advantages in wearable diagnostic and therapeutic integrated platforms, leveraging its excellent mechanical properties and hydrogel characteristics to support interstitial fluid collection, sustained drug release, and multimodal diagnostic and therapeutic integration.^84,85^ Combined with its nucleic acid loading capacity, CS also holds potential for integrating gene therapy into microneedle systems, offering new insights for the precise treatment of diabetic wounds.^86^

## 4. Structural designs of microneedles

### 4.1 Solid MNs

**Fig. 3:**
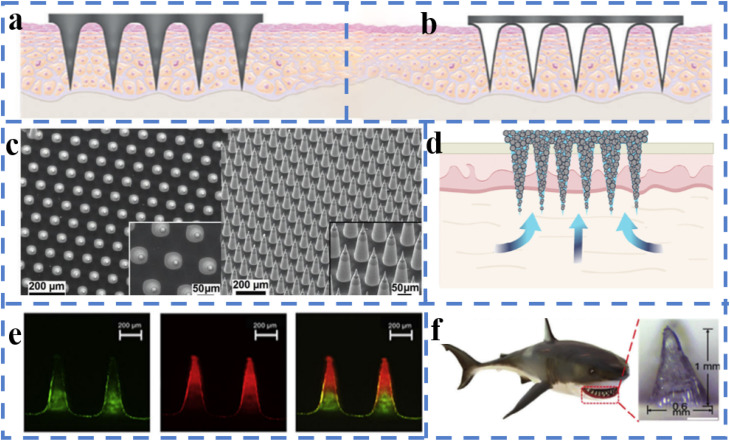
Structure of microneedles. (a) Solid microneedles. (b) Hollow microneedles.^36^ Reprinted with permission. Copyright 2023 Wiley-VCH GmbH. (c) Microneedle arrays.^52^ Reprinted with permission. Copyright 2022 Wiley-VCH GmbH. (d) Porous microneedles.^33^ Reprinted with permission. Copyright 2022 Wiley-VCH GmbH. (e) Multilayer microneedles.^48^ Reprinted with permission. Copyright 2022 Wiley-VCH GmbH. (f) Bioinspired structures.^106^ Reprinted with permission. Copyright 2024 Wiley-VCH GmbH.

Owing to their excellent mechanical strength and precise puncture capability, solid MNs have demonstrated significant potential in the local treatment of chronic diabetic wounds (Fig. 3a).^36^ In recent years, with the advancement of micro/nanofabrication technologies, the structure and functionality of solid MNs have significantly improved. For example, silicon-based microneedles fabricated *via* dry etching with xenon fluoride (XeF_2_) can achieve high density, controllable height (80–300 μm), and surface roughness, and their mechanical stability can be enhanced through titanium or chromium coating to maintain good integrity during skin penetration.^59^ In contrast, polymer-based MNs have attracted attention for their excellent biocompatibility and functional tunability, especially solid MNs constructed by 3D printing combined with conductive polymers, which not only have good puncture capabilities but also integrate biosensors to achieve real-time monitoring and feedback control of the wound status.^67^ Additionally, solid microneedles based on biodegradable hydrogels demonstrate excellent drug loading and release performance. GelMA/PEGDA composite microneedles utilize 3D printing and shrink molding technology to achieve structurally controllable multimodal drug delivery, making them suitable for scenarios requiring staged treatment in diabetic wounds.^54^ As microneedle manufacturing evolves toward cleanroom-free, low-cost production, solid microneedles are increasingly integrated into diagnostic and therapeutic platforms, providing robust tool support for intelligent, personalized treatment of diabetic wounds.^36^

### 4.2 Hollow MNs

Owing to their hollow structure, hollow microneedles (HMNs) can efficiently collect body fluids and deliver drugs, demonstrating unique advantages in the intelligent management of chronic diabetic wounds (Fig. 3b). Recent studies have shown that high-precision HMN arrays manufactured *via* laser drilling combined with a controlled negative pressure system can achieve continuous collection of interstitial fluid without relying on complex equipment. Additionally, by integrating electrochemical sensing modules, real-time monitoring of glucose and pH levels is enabled, providing critical support for closed-loop therapy.^87^ In local anti-infective therapy, the combination of hollow MNs with biodegradable materials significantly enhances the delivery efficiency and local stability of antifungal drugs while reducing systemic side effects.^88^ Additionally, hollow microneedles have been used to increase the accumulation efficiency of intradermal drug delivery in the lymphatic system, which has significant implications for targeted therapy in patients with diabetes complicated by inflammation or tumors.^89^ Furthermore, the integration of biomimetic design and smart responsive materials has driven the development of intelligent MN platforms that combine collection, monitoring, and drug release. For example, coral-like hollow microneedles can automatically adjust the release rate of antimicrobial drugs after the infection status is identified, enabling adaptive therapy.^90^ These advancements indicate that HMNs, as integrated diagnostic and therapeutic carriers, provide new technical pathways for dynamic regulation and personalized intervention in diabetic wounds.

### 4.3 Microneedle arrays

Microneedle arrays (MNAs) serve as highly customizable transdermal drug delivery and biosensing platforms, demonstrating diverse functions and significant therapeutic effects in the treatment of chronic diabetic wounds (Fig. 3c). This structure achieves direct contact with the dermis through the ordered arrangement of MN units capable of penetrating the skin barrier, providing an efficient pathway for biomarker detection and drug delivery. Hydrogel microneedle arrays have garnered particular attention, as they rapidly absorb interstitial fluid upon insertion into the skin to form continuous hydrogel channels, directly connecting the drug reservoir with the subcutaneous circulatory system. This significantly enhances the delivery efficiency of macromolecular drugs and overcomes the limitations of traditional transdermal delivery.^91^ Further structural optimization strategies, such as 3D-printed rigid shell-composite hydrogel core MN arrays, not only provide MNs with sufficient mechanical strength to penetrate the skin but also enable precise control of the drug release rate and depth by adjusting the hydrogel composition and MN geometry.^92^ In terms of biosignal detection, encoded microneedle arrays integrate photonic crystal barcodes with microneedles, enabling simultaneous enrichment and identification of multiple biomarkers within the skin. Their fluorescence intensity and reflection peak positions provide a dual recognition mechanism for high-throughput detection, making them valuable for assessing diabetes complicated by infection or inflammatory states.^93^ Additionally, glucose-responsive “enzyme-free” MN patches utilize borate hydrogels and silk protein to construct a stable scaffold, achieving excellent skin penetration performance while conferring acute and sustained insulin release capabilities. This provides the material foundation for developing a closed-loop intelligent insulin delivery system.^94^

### 4.4 Porous MNs

Microneedle arrays (MNAs) have been extensively explored and have demonstrated high adaptability and application potential in the treatment of chronic diabetic wounds because of their ability to achieve minimally invasive puncture, precise delivery, and biological sampling at the skin surface (Fig. 3d). MNAs effectively penetrate the stratum corneum through their micrometre-scale structures, avoiding the pain and infection risks associated with traditional puncture methods, and have thus emerged as an efficient and safe platform for drug delivery and diagnosis. In recent years, hydrogel MNs have been widely used for the collection and analysis of interstitial fluid because of their excellent biocompatibility and controllable expansion properties. The hydrogel channels formed after fluid absorption not only support the collection of wound exudate but also serve as an *in situ* analysis platform.^33^ This property has been further extended to diabetes wound healing monitoring, such as real-time feedback on glucose concentration and pH changes, providing a basis for personalized treatment.^95^ Additionally, the design flexibility of MN structures allows precise control of the drug release rate, depth, and duration by adjusting the array spacing, needle length, and material composition.^96^ For example, a rigid shell-composite hydrogel core structure combines sufficient mechanical strength with controlled-release functionality. In the context of diabetic wounds complicated by infection, microneedle arrays can also serve as carriers for antimicrobial therapy, delivering antifungal or antibiotic drugs *via* biodegradable materials to achieve long-term, stable local drug delivery.^48^ Further research indicates that integrating microneedles with biosensor systems can enable high-throughput detection of wound biomarkers. For example, microneedle arrays with integrated barcodes can visualize and quantify multiple metabolites or inflammatory factors, providing new pathways for early warning and intervention in diabetic infections.^98^ Notably, the application of MNs in regenerative medicine is driving their expansion into the fields of tissue functional reconstruction and injury repair. Their ability to deliver cytokines, stem cells, or nucleic acid-based drugs locally provides a multidimensional solution that combines structural support and biological regulation for the complex microenvironment of chronic diabetic wounds.^97^ Given the multiple pathological characteristics of diabetic wounds, such as high oxidative stress, low blood supply, and chronic inflammation, microneedle arrays can also be coupled with electronic systems to construct wearable closed-loop treatment systems, enabling synchronized drug delivery and real-time diagnostic feedback. Additionally, microneedle arrays are used to collect tissue fluid for auxiliary diagnosis of diseases such as cancer, as their highly sensitive liquid biopsy performance offers potential for monitoring multisystem diseases in diabetic patients. Although current MN materials are primarily synthetic polymers, the development of natural polymer composites offers new directions for long-term implantability and tissue compatibility.

### 4.5 Multilayer microneedles

Multilayer MN structures have emerged as a key direction in MN technology development in recent years because of their significant advantages in terms of drug loading capacity, release timing control, and functional integration, particularly demonstrating their unique potential in addressing the complex pathological environment of chronic diabetic wounds (Fig. 3e). On the basis of this design concept, Raphael *et al.* developed a high-density dissolvable microprojection array that enables multilayered vaccine loading within the MNs, allowing them to rapidly penetrate the superficial layers of the skin and release active components under needle-free, pain-free conditions. This configuration significantly enhances antigen diffusion efficiency and systemic immune responses,^99^ providing an important strategy for vaccine-based infection prevention in diabetic patients. Furthermore, Ning *et al.* proposed a dual-layer MN platform prepared *via* cryospray technology capable of simultaneously achieving effective insulin delivery and interstitial fluid collection and analysis, providing an integrated solution for dynamic monitoring and treatment response assessment at wound sites.^100^ In terms of inflammation relief, Chen *et al.* developed a lubricated double-layer soluble MN system that significantly reduces skin puncture damage by introducing a self-adhesive lubricating coating. They also employed a covalently bound double-layer drug release strategy to achieve multistage sustained release of anti-inflammatory drugs, thereby increasing treatment compliance and safety.^101^ Additionally, Zhou *et al.* developed a biomimetic microneedle chip with a multilayered microfluidic channel structure based on rolling microneedles, which can integrate a photonic crystal membrane for fluorescence-enhanced detection of inflammatory factors, demonstrating multifunctional synergistic capabilities in diabetes wound management.^102^ This type of multilayered MN system integrates spatial loading, responsive release, and real-time monitoring through structural partitioning, providing a high-performance, minimally invasive, and modular solution for the personalized treatment of diabetic wounds. Its scalability also offers broad prospects for the further development of intelligent microneedle platforms in the future.

### 4.6 Bioinspired structures

Bionic structures provide a wealth of design principles and manufacturing pathways for the functionalisation and performance optimization of MNs in the treatment of chronic wounds in patients with diabetes (Fig. 3f). The core of this approach lies in simulating the hierarchical structure, surface morphology, and mechanical gradients of natural tissues to achieve precise regulation of mechanical adaptation to the wound microenvironment, fluid management, and biological responses. The introduction of additive manufacturing technology not only enables the high-precision reproduction of complex biomimetic morphologies from the micrometer to millimeter scale but also facilitates the integration of channels, drainage grooves, or multichamber structures within microneedle arrays to enhance exudate guidance and drug distribution.^103^ Bionic design principles based on multiscale structures can be used to regulate the surface hydrophilicity/hydrophobicity, mechanical cushioning, and multifunctional surfaces of microneedles.^104^ In scenarios where mechanical compatibility and energy absorption at the wound site are prioritized, biomimetic composite materials and configurations provide excellent cushioning and stress-dispersing capabilities for MN patches, thereby reducing secondary damage caused by external forces and improving wearing comfort.^105^ For surface functionalization, 3D printing and surface micro/nanomanufacturing strategies enable the precise construction of biomimetic interfaces on microneedle surfaces. These structures can be used to guide exudate flow, enhance biomarker enrichment, or achieve optical indication functions, thereby aiding in the rapid identification of wound infections and triggering local drug delivery.^106^ Additionally, micro/nanoscale manufacturing methods provide the technical foundation for translating the mechanical–chemical synergistic effects of natural materials into artificial microneedle systems, enabling the integration of mechanical support, controlled-release chambers, and sensing units within a single patch. This drives the evolution of MNs from mere drug delivery devices to biomimetic therapeutic platforms capable of tissue regeneration and dynamic monitoring.^107^ Microneedle structural designs based on biomimicry and advanced manufacturing not only enhance wound affinity, drug utilization efficiency, and diagnostic/therapeutic precision but also provide scalable engineering pathways for staged, multimodal, and intelligent treatment of diabetic wounds.

## 5. Limitations of current therapies and advances in drug delivery

Owing to the complex etiology and pathogenesis of chronic wounds, current treatment options face considerable limitations regarding their suitability and efficacy. This complexity arises from the multifactorial nature of chronic wounds, which can be influenced by various factors, such as underlying health conditions, infections, circulatory issues, and the local wound environment.^108,109^ As a result, achieving the desired therapeutic outcome at every stage of the disease becomes a formidable challenge, ultimately contributing to a high rate of recurrence. Many existing treatments may be effective for certain aspects of wound healing but fail to address the comprehensive needs of patients, leading to prolonged healing times and repeated wound formation. Metal–organic framework-based transdermal drug delivery systems (MOF-TDDSs) were developed for diabetic patients because wound healing is severely disrupted by hyperglycemia, circulatory disorders, and infections, and the limitations of traditional treatments are significant. Its preparation technology involves hydrogels, microneedle patches and electrostatic spinning of nanofibers. Direct hydrogel preparation and *in situ* growth have their own advantages and disadvantages. Although metal–organic frameworks (MOFs) can optimize their performance, the dispersion problem needs to be solved; MN patch preparation relies on specific models and dispersing media; the bilayer structure has the potential to be constrained by drying defects; and electrostatically spun fibers can build porous structures to facilitate the release of MOFs but at a high cost. At the application level, hydrogels release ions and scavenge reactive oxygen species (ROS) to promote healing; microneedles break through physical barriers to achieve biofilm removal and tissue regeneration;^110–112^ electrostatically spun fibers synergize to promote wound healing; and microfluidic materials also show promise. Despite the outstanding advantages of MOF-TDDSs, the problems of stability, toxicity and cost are serious; thus, further optimization of the preparation technology and screening of the materials are necessary to accelerate the clinical translation process.

### 5.1 Innovative ways to deliver drugs

In recent years, significant progress has been made in the field of drug delivery, which offers promising avenues for improving wound care. Traditional methods of oral drug delivery, while advantageous in terms of high patient compliance and painlessness, often suffer from limitations related to the first-pass elimination effect. This phenomenon occurs when a drug is metabolized and rendered less effective by the liver before it reaches systemic circulation.^113,114^ Consequently, the bioavailability of many medications can be significantly reduced, limiting their overall effectiveness in treating chronic wounds. Advancements in drug delivery systems aim to overcome these challenges by increasing the absorption and efficacy of therapeutic agents. For example, novel methods such as transdermal patches, localized drug delivery, and advanced formulations can help bypass first-pass metabolism, allowing for more direct and efficient delivery of medications to the wound site. These innovative approaches not only improve the therapeutic concentration of drugs but also minimize side effects, increase patient comfort, and reduce overall treatment costs. By optimizing drug delivery, healthcare providers can more effectively target the specific pathophysiological mechanisms underlying chronic wounds, potentially leading to improved healing outcomes and lower recurrence rates. As research continues to evolve in this area, it holds the promise of transforming the management of chronic wounds, providing patients with more effective and tailored treatment options that address their unique healing needs. On the other hand, parenteral drug delivery methods, which involve the administration of medications through needle injection, offer notable advantages, particularly in ensuring rapid and targeted drug delivery. This approach allows quick absorption into the bloodstream, enabling healthcare providers to achieve effective therapeutic levels of drugs swiftly, which is especially critical in emergency situations or for managing acute conditions. As a result, parenteral methods have found widespread clinical applications, including in the treatment of infections, pain management, and various chronic diseases. However, despite these benefits, the acceptance of parenteral drug delivery among patients remains a significant challenge. Many individuals experience anxiety related to injections, commonly known as needle phobia. This fear can lead to considerable distress and reluctance to seek necessary medical treatments. Additionally, the pain associated with needle injections can deter patients from adhering to their prescribed medication regimens, particularly in cases requiring frequent or long-term injections. This combination of pain and anxiety can create barriers to effective treatment, limiting the potential for parenteral methods to be used more extensively in clinical practice. Patients may avoid necessary interventions, leading to suboptimal health outcomes and increased complications, particularly in chronic conditions where regular medication adherence is crucial. Furthermore, the logistical considerations of parenteral drug delivery can complicate its use. For example, the need for trained personnel to administer injections, the requirement for sterile conditions, and the potential for needle-related injuries all add layers of complexity to this delivery method. These factors can contribute to increased healthcare costs and resource allocation challenges. In light of these issues, interest in exploring alternative drug delivery systems that can provide the advantages of rapid absorption without the drawbacks of needle-based methods is increasing. Innovations such as transdermal patches, inhalable formulations, and microneedle technology are being developed to address patient acceptance while maintaining effective drug delivery. By improving the overall experience of patients, these alternatives have the potential to enhance treatment adherence and outcomes, making them promising candidates for future clinical applications.^115,116^

### 5.2 Research methodology

In terms of research methodology, both papers optimize the drug loading and release process by means of response surface methodology (RSM), which has a significant advantage over the traditional one-factor-one-at-a-time (OFAT) method, which is based on the principle of multivariate statistics and is able to comprehensively consider the effects of multiple factors and their interactions on response variables.^117,118^ By carefully designing the experimental protocol, an accurate mathematical model is constructed within a relatively small number of experiments so that the optimal experimental conditions can be precisely identified. This approach greatly improves the research efficiency; avoids the redundancy of the number of experiments due to the neglected interactions between factors in the OFAT method; significantly reduces the consumption of resources, including time, manpower, material and financial resources; and provides an efficient and scientific statistical analysis paradigm for optimization research of drug delivery systems.^119–121^

### 5.3 Mechanistic elucidation innovation

Recent advances in drug delivery and environmental nanotechnology have greatly enhanced the mechanistic understanding of how nanomaterials interact with pharmaceuticals under complex physicochemical conditions. Investigations into pH-dependent electrostatic interactions have shown that the ionization state of the drug and the surface charge of the nanoparticles are critical factors governing their adsorption behavior, thereby influencing the kinetics of drug loading and release.^122,123^ These findings underscore the importance of environmental parameters in modulating nanocarrier performance and guiding the design of stimuli-responsive systems.

In drug delivery applications, the surface chemistry, particle size, and charge distribution of nanomaterials have been demonstrated to play decisive roles in mediating interactions with biological systems, controlling drug-carrier affinity, and tuning release profiles.^124,125^ Such molecular-level insights are particularly relevant for creating targeted and pH-sensitive delivery systems capable of functioning in specialized microenvironments such as infected or tumor tissues. In parallel, substantial progress has been made in the use of environmental nanotechnology for the photocatalytic degradation of pharmaceutical pollutants. Magnetically separable semiconductor–zeolite composites, for example, have achieved high degradation efficiencies for antibiotic residues under visible-light irradiation, with photogenerated charge carriers identified as the dominant reactive species in direct Z-type transfer pathways.

Heterojunction engineering has emerged as a powerful strategy for enhancing photocatalytic performance, with visible-light-active composites benefiting from optimized band alignment and synergistic cocatalyst effects. The Z-scheme pathway has proven effective in promoting charge separation, enabling the breakdown of structurally stable pharmaceutical compounds. The composite structure and dopant ratio have been shown to markedly influence the degradation efficiency of various semiconductor–support systems. The application of kinetic modeling, particularly the Hinshelwood equation, provides a robust framework for describing reaction order, elucidating mineralization pathways, and optimizing system performance. Integrating material design, photocatalytic behavior, and mechanistic modeling offers a rational approach to advancing nanocarriers for both therapeutic and environmental applications, bridging the gap between laboratory synthesis and real-world deployment.^126,127^

### 5.4 MOFs in antimicrobial and drug delivery applications

Peng and focused on the application of metal–organic frameworks (MOFs) in the field of antimicrobial therapy, which exhibit unique advantages in antimicrobial therapy because of their high specific surface area, tunable structure, and good biodegradability. The antimicrobial mechanisms of metal–organic frameworks (MOFs) based on different metal ions, including metal ion release and reactive oxygen species (ROS) generation, are systematically discussed.^128,129^ For example, zinc–benzene-1,3,5-tricarboxylate (Zn–BTC) facilitates sustained Zn(ii) release to enhance wound healing, whereas the zirconium-based framework UiO-66 serves as a drug carrier for acne treatment. Moreover, problems that need to be solved before the clinical application of MOFs, such as the construction of a chronic wound model, the control of metal ion release, the optimization of biosafety and targeting, *etc.*, have also been identified, which provides a comprehensive reference for the further development of MOFs in the field of antimicrobials. Ma's team focused on the application of UiO-based MOFs in breast cancer treatment. The advantages of UiO-MOFs, such as good biocompatibility, high specific surface area and stability, are presented. They are used in different modalities of breast cancer treatment, such as photodynamic therapy (PDT), chemotherapy (CT), positron emission tomography (PET), chemodynamic therapy (CDT) and combination therapy.^130–134^ In PDT, UiO-66 can be loaded with photosensitizers to improve its therapeutic effect; in CT, it can be used as a drug carrier to improve drug delivery; and in combination therapy, it can combine the advantages of different therapeutic modalities to overcome the limitations of single therapy. In combination therapy, UiO-66 can combine the advantages of different therapeutic modalities to overcome the limitations of single therapy. However, some UiO-66 MOFs have poor alkali resistance and insufficient *in vivo* studies, which highlights the direction of subsequent research. Lu and colleagues focused on the biomedical applications of MIL-100(Fe) and MIL-101(Fe). These two types of MOFs have unique crystal structures, high specific surface areas, adjustable pore sizes and good chemical stability. In drug delivery, they have good loading and release capabilities for NSAIDs and ophthalmic drugs; in antimicrobial therapy, they exhibit broad-spectrum antimicrobial activity against a wide range of bacteria; and they also play important roles in multiple modes of cancer treatment (chemotherapy, CDT, photothermal therapy (PTT), PDT, immunotherapy, *etc.*). However, there are challenges in its stability, industrial production and clinical studies, which require further research for improvement. Zhou's team investigated the use of MOFs loaded with H_2_O_2_-related substances in tumor iron death therapy. First, the iron death mechanism and substances that can increase intracellular H_2_O_2_ levels, such as alkaloids and terpenoids, were introduced. The strategies of different metal ion (Fe, Cu, Mn, *etc.*)-based MOFs loaded with related substances to promote iron death, such as Fe ion-MOFs loaded with GOx or DOX, are described below. The experiments revealed that these MOFs are effective in tumor therapy, but there are deficiencies in terms of synthesis methods, drug delivery efficiency, safety and *in vivo* studies, which provides a direction for improvement in subsequent studies. Peng's team reviewed the progress of Ti-based MOFs in biomedical applications, which have unique structural and performance advantages and potential applications in bioimaging, drug delivery and cancer therapy. In bioimaging, they can be used as contrast agents to improve the imaging effect; in drug delivery, they can effectively load and release drugs; and in cancer therapy, they can show certain therapeutic effects in photodynamic therapy and chemodynamic therapy by combining them with other materials or therapeutic modalities, but they also face a number of problems, such as optimization of the stability and biocompatibility of the materials, which need to be solved.

Bai and colleagues focused on the progress of Fe(iii)Fe(ii)-MPNs in biomedical applications. Fe(iii)Fe(ii)-MPNs have special structures and properties and have shown promising applications in antimicrobial, antioxidant, and anticancer therapies. Antimicrobial agents can inhibit bacterial growth through mechanisms such as releasing iron ions and generating reactive oxygen species; antioxidants can scavenge free radicals; and anticancer agents can be used as drug carriers or play an anticancer role by inducing apoptosis. Moreover, its challenges in terms of synthesis complexity and biosafety assessment are also noted, which provides a reference for subsequent research.^135–138^

## 6. Applications of microneedle patches in diabetic wound healing

In the context of the continuous development of microneedling technology, its potential in the treatment of diabetes-related diseases is particularly prominent, in addition to enhancing the convenience and efficacy of treatment. Diabetic patients often face difficulties in wound healing, mainly due to impaired immune function and poor blood circulation caused by hyperglycemia.^139,140^ Owing to its precise drug delivery, microneedle technology is able to address these problems during treatment. Through the optimization of MN design and function, a multifaceted treatment protocol can be developed that can reduce blood glucose levels, inhibit infection, eliminate reactive oxygen species, and promote wound healing. This multifaceted MN system not only directly enhances the wound healing environment but also promotes innovations and advancements in diabetic wound therapy. Consequently, the application of microneedle technology in diabetic wound treatment is emerging as a therapeutic tool with significant potential.^141–143^

### 6.1 Blood glucose regulation

**Fig. 4:**
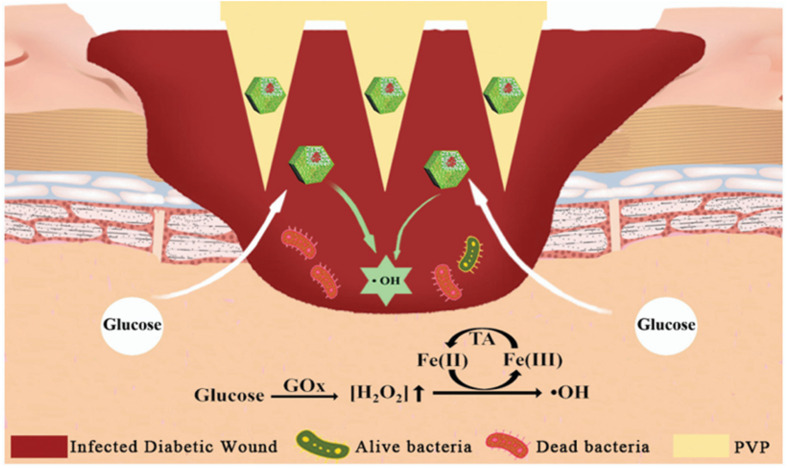
Penetration of MOF-based MNs into infected diabetic wounds to release antibacterial particles for the treatment of infected diabetic wounds.^144^ Reprinted with permission. Copyright 2022 Royal Society of Chemistry.

Diabetic wounds are characterized by uncontrolled blood glucose levels and vulnerability to bacterial infection. The process of wound healing can be accelerated by concurrently reducing glucose levels and protecting the wound from bacterial infection (Fig. 4). These findings suggest that glucose oxidase (Gox)-involved systems are promising candidates for the treatment of infected diabetic wounds.^145,146^ MNs have the capacity to deliver various types of drugs or carriers to specific sites. The most common treatment for skin infections is the use of gels, creams and ointments that contain antimicrobial agents. However, these methods are often limited by the inefficient penetration of antimicrobial agents through extracellular polymeric substances into biofilms. MNs, however, can efficiently penetrate biofilms and release antimicrobial agents, which makes the use of MNs an ideal technique for treating infected wounds. In addition, various molecules or particles have been used in combination with MNs for stimuli-responsive antimicrobial therapy, including local pH changes or heating by infrared radiation. The combination of MOFs and MNs for glucose-responsive therapy of infected diabetic wounds is reported for the first time in this article. The construction and encapsulation of a graded porous MOF (GOx@Fe-ZIF-TA) into soluble polyvinylpyrrolidone (PVP) has enabled the fabrication of stimuli-responsive MNs for the treatment of infected diabetic wounds. This application of a graded porous MOF represents a novel approach for excess glucose consumption, with the potential to protect infected diabetic wounds from bacterial infection.^147^

This combination of microneedle (MN)-based technology and stimuli-responsive materials provides a novel solution for the treatment of diabetic wounds. In addition, this study offers theoretical justification and experimental support for the further improvement of wound healing. The integration of a GOx complex and a porous metal–organic framework (MOF) within the microneedle facilitates the local consumption of excessive glucose in diabetic wounds, thereby reducing blood glucose levels and effectively mitigating inflammatory responses triggered by excess glucose. The design of the graded porous MOF material allows the microneedle to better penetrate the wound area, releasing both GOx and antimicrobial components to form a self-reinforcing therapeutic mechanism. On the one hand, GOx generates hydrogen peroxide (H_2_O_2_) and gluconic acid through glucose oxidation to generate a localized acidic environment, which effectively inhibits the growth of bacteria. In addition, the Fe(ii)/Fe(iii) redox reaction further promotes the generation of antibacterial reactions, thus enhancing the therapeutic effect of the MN system. The primary benefit of this design is that it is highly targeted, enabling efficient action at the wound site with a reduced impact on healthy tissue. In addition, the use of stimulus-responsive materials allows the microneedle to intelligently adjust its release rate according to the glucose concentration in the wound and changes in the local environment, thus enabling precise and sustained treatment. This composite microneedle system for diabetic wounds has the potential to address the challenge of drug resistance that may emerge in conventional antibiotic therapy, thereby offering a more convenient and personalized treatment option for diabetic patients.^148–151^

In addition to the reduction in glucose levels at the wound site to promote healing, diabetic wounds often face another serious problem in the form of bacterial infection. Owing to the suppression of the immune system in diabetic patients, the risk of wound infection is significantly increased, and bacteria not only slow the healing process but can also lead to more serious complications. Therefore, in addition to controlling blood glucose levels, the effective administration of antimicrobial therapy is crucial for the repair of diabetic wounds. The innovative design of MN technology offers a promising solution to address this issue by expanding the application of MNs in wound treatment. The integration of antimicrobial components with stimulus–response mechanisms ensures the precise delivery of drugs and the sustained delivery of antimicrobial protection at the wound site, thereby enhancing the therapeutic effect in multiple dimensions. In the subsequent section, we explore the innovative design of MNs to combat bacterial infections in diabetic wounds and further advance the wound healing process.

### 6.2 Antimicrobial therapy

In the context of diabetic wound healing, the management of blood sugar levels is paramount, with the prevention of bacterial infection being a pivotal factor in accelerating the process of wound healing. The immune system of diabetic patients is often compromised, rendering their wounds vulnerable to bacterial infiltration, particularly by common pathogens such as *Staphylococcus aureus* (*S. aureus*). Consequently, the inhibition of bacterial growth and the mitigation of infections have emerged as crucial strategies to promote diabetic wound healing. In this context, researchers have developed a variety of innovative approaches, among which the use of microneedle technology to inhibit bacterial infections has emerged as a particularly effective treatment. By combining antimicrobial agents in MNs or utilizing photosensitive materials, MNs not only precisely release antimicrobial components but also provide a sustained local antimicrobial effect at the wound site. These MNs have been shown to target the bacterial community surrounding wounds, thereby effectively inhibiting bacterial growth and promoting wound healing.^110,158–160^

**Fig. 5:**
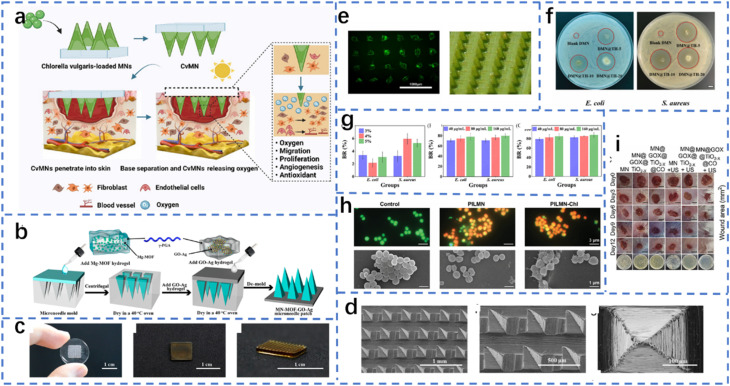
(a) Schematic of the CvMN-based sunlight-triggered oxygen-generating system for enhanced diabetic wound healing.^152^ Reprinted with permission. Copyright 2023 American Chemical Society. (b) Schematic of the synthesis of MN-MOF-GO-Ag. (c) Photographic images of the polydimethylsiloxane (PDMS) MN mold and top-down and isometric photographic images of MN-MOF-GO-Ag. (d) SEM images of MN-MOF-GO-Ag.^153^ Reprinted with permission. Copyright 2021 American Chemical Society. (e) Fluorescence images of agarose hydrogels and photographs of CvMN tips after the application of CvMN.^152^ (f) Digital photographs of *Staphylococcus aureus* colonies and *Escherichia coli* after incubation with different DMNs for 12 h.^154^ Reprinted with permission. Copyright 2023 Wiley-VCH GmbH. (g) Bacterial inhibition (BR) of MN-treated bacterial suspensions with different concentrations of HA, A-GNCs and A-GNCs.^155^ Reprinted with permission. Copyright 2024 Wiley-VCH GmbH. (h) Live/dead staining and SEM images of MRSA after 4 h of culture with PILMNs or PILMN-chl.^156^ Reprinted with permission. Copyright 2024 Wiley-VCH GmbH. (i) Images of diabetic wounds infected with *S. aureus* at 0, 3, 6, 9 and 12 days after treatment.^157^ Reprinted with permission. Copyright 2025 Wiley-VCH GmbH.

The process of wound healing in individuals suffering from diabetes is influenced by a multitude of factors, with bacterial infection at the wound site representing a significant challenge. Fig. 5a illustrates the sunlight-triggered oxygen generation system based on microneedles (CvMNs) that has been devised, which addresses the issue of inadequate oxygen supply in diabetic wounds through a meticulously designed microneedle platform. Concurrently, it delivers reactive oxygen species in a minimally invasive manner to promote wound healing. CvMN not only continuously generates oxygen under light but also effectively inhibits periwound inflammation through the effects of antioxidants and micronutrients, thus accelerating angiogenesis and fibroblast proliferation. The inflammatory response, which in turn, accelerates angiogenesis and fibroblast proliferation. This design exploits the transdermal delivery of MNs while addressing the common problems of hypoxia and bacterial infection in diabetic wounds. Fig. 5e further validates the ability to isolate CvMNs. In the agarose hydrogel model, the substrate layer dissolved rapidly, allowing the GelMA tip containing the active ingredient to remain stable on the hydrogel surface, thus effectively ensuring oxygen permeability. This experiment not only verified the efficient separation ability of the microneedle system but also demonstrated its good adaptability to the skin in practical applications, revealing the great potential of microneedles as therapeutic tools for diabetic wounds. Fig. 5h shows our developed drug-loaded microneedle system (PILMN-Chl), which integrates photosynthesis to generate stabilized oxygen and delivers it directly to the wound area *via* the microneedle. This innovative approach has been demonstrated to significantly reduce bacterial infections and promote wound healing by inhibiting the growth of methicillin-resistant *Staphylococcus aureus* (MRSA) and other pathogenic bacteria. PILMN-Chl exhibited significant antibacterial activity, and after a 4 hours coculture experiment, the bacterial cells underwent significant morphological changes. The cell membrane was damaged and exhibited irregularity and collapse, further confirming its promising application in the treatment of chronic diabetic wounds. Fig. 5d shows an ultrasound-responsive microneedle (MN@GOX@TiO_2_-X@CO) system, which is capable of efficiently releasing carbon monoxide (CO) in response to ultrasound stimulation to control diabetic wound infections and promote healing. This system has been designed for the treatment of diabetic wounds and not only exhibits greater antimicrobial efficacy than conventional treatments but also significantly accelerates the wound healing process. The targeted release of CO within a wound induced by ultrasound stimulation directly affects infected wound tissue, thereby slowing the growth of pathogenic bacteria and promoting wound tissue repair and regeneration. As demonstrated in Fig. 5g, the microneedle drug-carrying system exhibits an innovative design through the combination of a hyaluronic acid (HA) substrate and aminobenzeneboronic acid-modified gold nanoclusters (A-GNCs). This innovative design has been shown to disrupt bacterial cell membranes and inhibit their growth while also providing real-time monitoring through fluorescence emission under ultraviolet radiation. This, in turn, ensures the precision of drug delivery and antimicrobial efficacy. In *in vitro* experiments, A-GNC MNs demonstrated good antimicrobial effects and significantly improved the healing speed of diabetic wounds, which demonstrates the multiple advantages of MNs in the treatment of diabetic wounds, especially their unique value in controlling infections and promoting wound healing. The multifunctional dual-layer microneedle (DMN) system, which incorporates drug-carrying microneedles of antimicrobial drugs and growth factors, is demonstrated in Fig. 5f. The precise release of drugs is achieved through a gelatinase-responsive mechanism, and the bilayer microneedle is able to promote angiogenesis and tissue repair along with antimicrobial activity. In a rat diabetic wound model, the DMN system not only significantly improved local angiogenesis and collagen deposition but also accelerated wound healing, further demonstrating the innovative application of MNs in the treatment of chronic diabetic wounds.^161–163^

As demonstrated by previous studies, microneedle technology has considerable potential in the field of diabetic wound therapy. The design of MNs allows for the precise delivery of drugs, clearance of infections, and resolution of critical issues in diabetic wound healing through a number of innovative methods. However, notably, the process of diabetic wound healing is influenced by various factors, including bacterial infections and oxygen supply problems. In addition, excessive accumulation of reactive oxygen species plays a significant role in poor long-term diabetic wound healing. Consequently, future research endeavors will concentrate on the utilization of microneedling techniques to target and remove reactive oxygen species from wounds, with the aim of enhancing wound healing and mitigating chronic wound challenges in diabetic patients.

Excessive ROS production represents a significant impediment to diabetic wound healing, as elevated ROS levels have been demonstrated to inhibit skin regeneration and trigger a sustained inflammatory response, thereby exacerbating tissue damage and delaying the wound healing process. This effect is particularly pronounced in diabetic patients because of immune dysfunction and metabolic abnormalities, resulting in an accumulation of ROS that often exceeds the body's capacity for self-clearance. Consequently, the development of effective strategies for the regulation of ROS generation and removal has emerged as a pivotal area of research in the field of chronic wound healing in diabetes. Owing to its distinctive structure and function, the microneedle system offers a novel and highly effective therapeutic approach. It facilitates the local and precise delivery of active ingredients while simultaneously regulating ROS levels through direct action on the wound microenvironment. In contrast to conventional systemic drug therapies, microneedling is capable of delivering active ingredients in a localized manner without eliciting systemic adverse effects, thereby extinguishing excess ROS and attenuating damage to healthy tissue while expediting the healing process.^169–171^

The innovation of SeC@PA microneedles is predicated on two mechanisms of action: first, the enhancement of ROS production through the depletion of endogenous glutathione (GSH), a process that has been shown to inhibit bacterial infections, particularly in the context of biofilms. Second, the prevention of the cellular damage and tissue inflammation triggered by excessive ROS involves regulating the clearance of ROS at low GSH levels. In the context of chronic diabetic wounds, the low-GSH environment facilitates the unique therapeutic advantages of SeC@PA MNs. SeC@PA microneedles exhibit adaptive responses to changes in the microenvironment, regulating the concentration of ROS at different stages and effectively avoiding the adverse effects caused by excessive ROS, thereby promoting wound healing.

The CMSP-MNs microneedle design offers a unique advantage in its capacity to address bacterial infections and oxidative stress induced by ROS overproduction.^55^ Copper–metal–sulfide (CMS) nanoparticle-loaded MNs facilitate not only the effective generation of ROS and bacterial inactivation during the bactericidal process but also the scavenging of excessive ROS in wounds. These compounds can also alleviate inflammation caused by hypoxia through the action of PDAs. The multifunctionality of PDAs increases the tunability of MNs, not only by generating ROS through catalytic reactions but also by acting as antioxidants to reduce the adverse effects of ROS when necessary. Consequently, the CMSP-MNs system reveals novel avenues for the application of microneedle technology in the domain of antimicrobial and anti-inflammatory therapies, particularly in the context of wound treatment for diabetic patients. In this scenario, precise regulation of the ROS level in the microenvironment can be achieved to circumvent excessive oxidative stress.

The design of the strontium polyphenol network (SrC-MPN) microneedle patch represents a novel approach for the management of oxidative stress and the promotion of angiogenesis through the release of chlorogenic acid (CGA), which functions as a scavenger of excess ROS. Concurrently, the release of strontium ions stimulates angiogenesis at the wound site. Angiogenesis is an integral component of the wound healing process, as it provides the necessary supply of nutrients and oxygen to facilitate the repair process. Therefore, SrC-MPN microneedling not only improves local inflammation by removing ROS but also further accelerates healing by promoting angiogenesis.

In contrast, hydrogen-loaded MNs (MN-MgH_2_) have been shown to be effective therapeutic solutions for treating diabetic wounds. The unique bidirectional response mechanism of these microneedles has been demonstrated to reduce the level of ROS, thereby improving the local oxidative stress environment of the wound and reducing cellular damage. This, in turn, has been shown to promote wound healing. The release of Mg^2+^ is capable of promoting the polarization of M2-type macrophages, which in turn facilitates tissue repair and neovascularization (B1). The dual mechanism of action of the MN-MgH_2_ MNs results in a reduction in ROS levels in the wound and an increase in the immune response, thereby creating an ideal healing microenvironment. The design of this microneedle system demonstrates the strong potential of microneedles for preventing oxidative stress, especially in the treatment of wounds associated with chronic diseases such as diabetes mellitus, to effectively regulate the local redox state, leading to rapid wound healing.

The innovative application of MN technology for scavenging ROS and regulating oxidative stress has been shown to significantly enhance the healing of the microenvironment of diabetic wounds.^23,171,172^ Through precise modulation of ROS production and removal, microneedling has been demonstrated to effectively reduce inflammation, stimulate cellular repair and angiogenesis, and expedite the healing process of chronic diabetic wounds. These innovative MN systems offer more personalized treatment options and provide new hope for wound treatment in diabetic patients, especially for chronic wounds, which require long-term management. The potential for clinical application is significant.

In the preceding discussion, an exhaustive analysis was conducted on the innovations of microneedle technology in the regulation of ROS, with a particular focus on the promotion of wound healing through the precise modulation of oxidative stress. However, the delayed healing of diabetic wounds is not solely attributed to excess ROS; the persistence of a chronic inflammatory response plays a pivotal role. Chronic inflammation not only exacerbates oxidative stress but also inhibits fibroblast proliferation, angiogenesis and tissue remodeling, consequently delaying the healing process. Thus, effectively managing the local inflammatory response in wound therapy is imperative. In the following section, the innovative role of MNs in the anti-inflammatory treatment of diabetic wounds is explored. By modulating ROS levels, MNs alleviate the inflammatory response and help inhibit the excessive inflammatory response by promoting the release of anti-inflammatory cytokines and modulating the immune response. This has been demonstrated to effectively improve the healing environment of diabetic wounds. The potential for microneedle technology in anti-inflammatory therapy is evident, and this potential can be further enhanced through the design of novel microneedle systems and functional optimization. The following discussion introduces several MN-based anti-inflammatory therapeutic strategies and explores the prospects for their clinical application in the treatment of diabetic wounds.

### 6.3 Anti-inflammatory mechanisms

In addition to the ongoing development of microneedling technology, which has achieved significant progress in reducing oxidative stress (ROS) and modulating immune responses, another important innovation is the promotion of diabetic wound healing through the precise delivery of growth factors.^175,176^ Growth factors play crucial roles in tissue repair by stimulating cell proliferation, angiogenesis and tissue regeneration, thereby accelerating wound healing. In diabetic wounds, deficiency or dysregulation of these factors frequently results in delayed healing, underscoring the critical importance of effective delivery of growth factors in enhancing the rate of wound healing. Owing to their unique drug delivery capabilities, microneedle systems have emerged as a significant means of achieving this objective. In the subsequent section, we delve into the innovations of microneedles in augmenting growth factor delivery and further elaborate on their potential in promoting diabetic wound healing.

In recent years, a growing body of research has demonstrated the significant potential of microneedle technology as an innovative drug delivery system for the treatment of diabetic trauma. Fig. 7a shows a battery-free, multicomponent drug-carrying electronic microneedle (mD-eMN) system for efficient drug delivery, which integrates a remodeled metal microneedle and a flexible friction electrical nanogenerator (TENG). Pulsed electrons, synergized by TENGs, are able to rapidly release drugs to inflammatory sites (ISDs), thereby increasing drug penetration, modulating immune responses, and effectively reducing local inflammation. Compared with conventional electrical stimulation (ES) or chemotherapy alone, the mD-eMN system significantly reduced skin inflammation in psoriasis models, validating its potential for clinical application. Fig. 7a further shows the excellent penetration ability of the system on the hard skin of pigs, highlighting its broad application prospects.

**Fig. 6:**
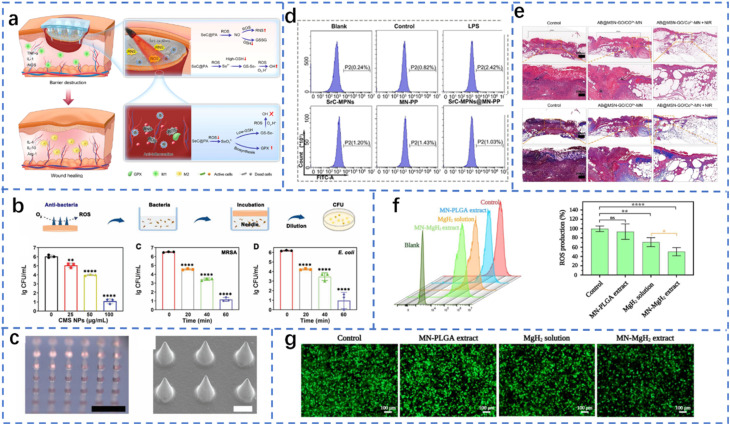
(a) Schematic of the fabrication process of the SeC@PA MN bandage and its wound healing mechanism.^164^ Reprinted with permission. Copyright 2023 Springer Nature. (b) *In vitro* antibacterial activity of CMSP-MNs. Bacterial inactivation efficiency of different doses of CMS NPs against MRSA and *E. coli*.^165^ Reprinted with permission. Copyright 2023 Wiley-VCH GmbH. (c) Photograph and SEM image of CMSP-MNs (scale bars: 1 mm and 200 μm, respectively).^165^ (d) Flow cytometric analysis of ROS production in Raw264.7 cells treated with different MN formulations.^166^ Reprinted with permission. Copyright 2024 Elsevier. (e) H&E and Masson's trichrome staining of diabetic wounds on day 9 for each treatment group.^167^ Reprinted with permission. Copyright 2024 Elsevier. (f) ROS fluorescence staining of Raw264.7 cells after different treatments and the corresponding statistical analysis.^168^ Reprinted with permission. Copyright 2023 Elsevier. (g) ROS assay in Raw264.7 cells after 24 hours of treatment with MN-PLGA extract, MN-MgH_2_ solution, or control medium. ROS were stained with DCFH-DA (green fluorescence).^168^

**Fig. 7:**
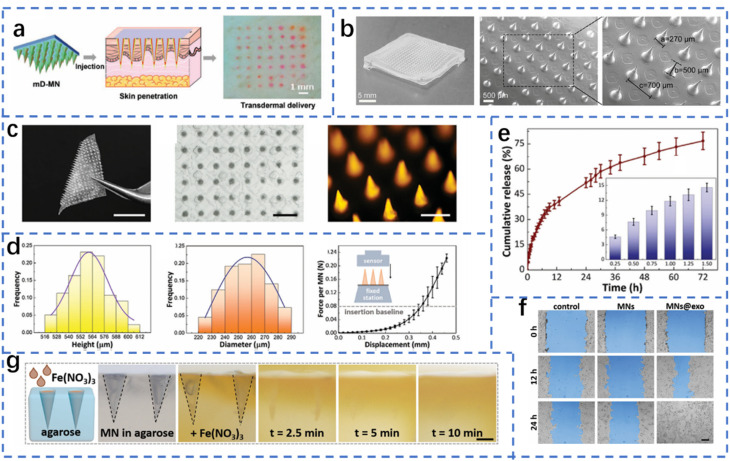
(a) Schematic of mD-MN-mediated transdermal drug delivery and digital image of porcine skin after application of the mD-MN patch (PVP labeled with coumarin 6, carbomer with rhodamine B).^173^ Reprinted with permission. Copyright 2023 Wiley-VCH GmbH. (b) High-resolution images of the CTH@MN patch morphology and SEM microstructure.^168^ Reprinted with permission. Copyright 2023 Elsevier. (c) Optical and fluorescence images of the indwelling MN patch.^174^ (d) Distribution of the needle tip height (*n* = 177), base diameter (*n* = 177), and stress tolerance of the needle tips under pressure.^174^ (e) Cumulative release of fluorescent nanoparticles from the needle tip into PBS over 3 days.^174^ (f) Optical image of the NIH/3T3 cell scratch assay with pseudocolor applied.^174^ (g) Schematic and time-lapse images of needle tip interactions with a Fe(NO_3_)_3_ solution in an agarose block.^174^ Reprinted with permission. Copyright 2023 Wiley-VCH GmbH.

Furthermore, Fig. 7b shows an antioxidant, anti-inflammatory, and antiaging CeO_2_@Tau nanoparticle developed by loading taurine (Tau) into cerium dioxide (CeO_2_). These nanoparticles were encapsulated in a gelatin methacryloyl (GelMA) hydrogel to form the CeO_2_@Tau@hydrogel@microneedle (CTH@MN) system. The CTH@MN system significantly improved penetration into diabetic wounds and was able to release taurine precisely and efficiently, thereby effectively promoting wound healing. The SEM image in Fig. 7b shows the precise structure of the MN array, enhancing its ability to puncture the skin. Further experiments demonstrated that the CTH@MN system had significant therapeutic effects on diabetic wounds by inhibiting the ROS/NF-κB signaling pathway and significantly reducing oxidative damage and inflammatory responses. Furthermore, the CTH@MN system has been shown to create an immune microenvironment conducive to tissue repair by activating autophagy, particularly in a diabetic mouse wound model. In addition, CTH@MN has been demonstrated to significantly accelerate wound healing by regulating the oxidative-inflammatory-aging (OXI-Aging) pathological axis.

Fig. 7c–g illustrates the pioneering microneedle (MN) design pioneered by Zhao *et al.*, which employs a combination of 3D transfer technology and template replication to achieve optimal bionic adaptability.^174^ This sophisticated design facilitates the precise encapsulation of exosomes derived from mesenchymal stem cells (MSCs). The tip of the microneedle is composed of a tunable polyester alcohol (PVA) hydrogel material that can be dynamically adjusted to the mechanical strength of the tissue during diabetic ulcer treatment (Fig. 7f). This innovative design enhances the mechanical properties of the PVA hydrogel by introducing the Hoffmeister effect, which allows the MN to adapt to different tissue strengths and ensures effective skin penetration (Fig. 7g).^174^ As demonstrated in Fig. 7c, following insertion into the skin, the microneedle tip undergoes a softening process, thereby optimizing its compatibility with the wound tissue and reducing patient discomfort. Fig. 7d further illustrates the softening of the MNs postpenetration into the skin and their effective integration with the wound environment. Fig. 7e subsequently shows the pivotal role of intracellular vesicles (exosomes) in promoting cell migration. As demonstrated in Fig. 5f and g, the microneedle system was shown to enhance the healing of diabetic wounds in a diabetic mouse wound model. This study revealed that MSC-derived exosome therapy promoted angiogenesis and effectively stimulated the activity of fibroblasts and macrophages, thereby improving the wound microenvironment and accelerating wound healing. The combination of advanced microneedling technology and the biological activity of exosome therapy significantly enhanced the effectiveness of diabetic wound treatment. The innovation of the microneedle system not only compensates for the shortcomings of traditional wound treatment methods but also significantly improves the healing speed and quality of diabetic wounds through precise drug release and cellular repair mechanisms. Owing to their efficient delivery ability and biocompatibility, MNs have great potential for application in complex wound treatments, such as diabetic ulcers.

Despite the encouraging outcomes demonstrated by microneedle systems in enhancing diabetic wound healing through the modulation of redox reactions and immune mechanisms, among other pathways, the process of wound healing encompasses a multitude of complex pathways. The pivotal role of growth factors in diabetic wound repair has garnered significant recognition, as their intervention has been shown to markedly accelerate wound healing by promoting cell proliferation and accelerating angiogenesis and tissue repair. Consequently, the integration of MN technology with growth factor delivery offers a novel direction for investigating diabetic wound healing. The subsequent discussion will explore the potential of microneedling to increase growth factor delivery, with the aim of further improving diabetic wound healing.

### 6.4 Growth factor stimulation

In the preceding discussion, the focus was on the anti-inflammatory innovations of microneedle technology and its contribution to diabetic wound healing. By effectively modulating oxidative stress and inflammatory responses, MN systems significantly facilitate the process of wound repair. However, wound healing is not only dependent on controlling the inflammatory response; factors such as growth factors, cellular repair and angiogenesis also play crucial roles. In this context, composite innovations in microneedling, particularly in conjunction with bioactives and cytokines, present a novel approach with the potential to further expedite diabetic wound healing.

In recent years, interest in microneedle technology as a promising innovation in the field of drug delivery for diabetic trauma therapy has increased. Fig. 6a shows a novel stem cell sphere-loaded microneedle (MN@SP) patch, which was prepared through the use of microfluidic template technology. This method has the capacity to generate stem cell spheres (SPs) of uniform size *in situ* through the precise manipulation of the microfluidic environment. The stem cell spheres (SPs) thus produced have been shown to exhibit excellent cell viability and to express features related to the extracellular matrix and angiogenesis. Once loaded into MNs, these SPs can be used to deliver and exchange a variety of active substances, thereby promoting angiogenesis, collagen deposition and tissue repair in diabetic wounds. The MN@SP patch thus shows great potential in the treatment of diabetic wounds and provides a new direction for subsequent research.^177^

**Fig. 8:**
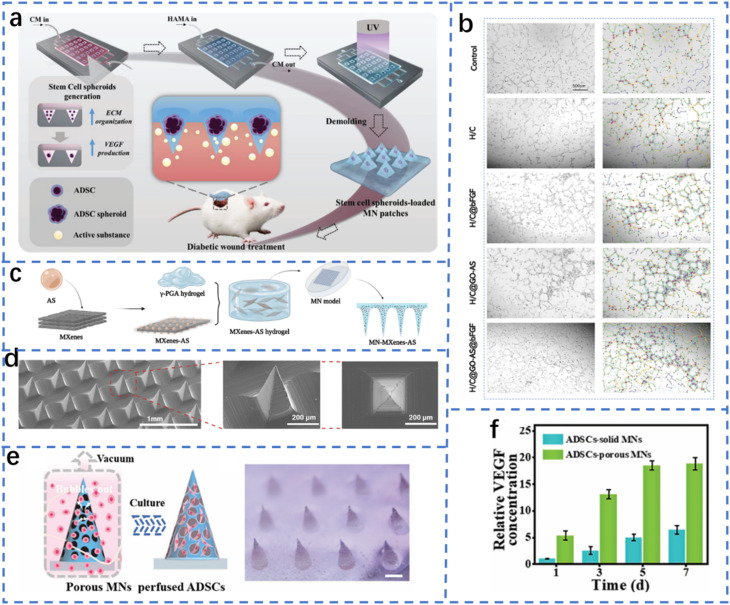
(a) Fabrication of the microfluidic-templated MN@SP patch and its application in diabetic wound healing.^177^ Reprinted with permission. Copyright 2023 Wiley-VCH GmbH. (b) Tube formation assay with HUVECs, showing endothelial cell growth in each group.^178^ Reprinted with permission. Copyright 2023 Elsevier. (c) Schematic illustration of the synthesis of the MN-MXene-AS composite.^179^ (d) SEM images of the MN-MXene-AS patch from various angles.^179^ Reprinted with permission. Copyright 2022 Springer Nature. (e) Schematic and optical images of porous MN arrays loaded with ADSCs (scale bar, 400 μm). (f) Proliferation of ADSCs on porous MNs quantified by statistical analysis and fluorescence staining at 1, 3, 5, and 7 days.^180^ Reprinted with permission. Copyright 2024 Wiley-VCH GmbH.

As demonstrated in Fig. 8b, the innovative design of a multifunctional hyaluronan methacrylate (HAMA)/carboxymethyl chitosan (CMCS) core–shell MN patch combines the degrading action of hyaluronidase and the sustained release of growth factors to significantly improve diabetic wound healing. The action of hyaluronidase, which is responsible for the degradation of the patch, has two notable effects. First, it promotes the release of graphene oxide, and second, it triggers potent antimicrobial activity through posttranscriptional regulation and physical cleavage. The continuous release of basic fibroblast growth factor (bFGF) in the microneedle accelerates angiogenesis, collagen synthesis and immunomodulation. These effects synergize with biodegradable HAMA to significantly improve the efficiency of wound healing. The experiments in Fig. 8b further validated the effectiveness of the system in promoting tube formation, fully demonstrating the comprehensive advantages of MNs in wound repair while reflecting their strong potential in biological functions.^178^

As Fig. 8c illustrates, the innovative application of Ti-2C MXenes combined with poly-γ-glutamic acid (γ-PGA) hydrogels in a microneedle (MN-MXenes-AS) has been developed. This microneedle system was designed to promote cell proliferation and modulate angiogenesis by loading cumene glycosides into MXene-enhanced hydrogels. A comprehensive evaluation of the characteristics and mechanical strength of the MNs was conducted, which revealed that MXenes significantly enhanced the mechanical strength of the MNs, whereas the γ-PGA hydrogel provided an ideal moist environment for wound healing. Importantly, the experiments were conducted on a diabetic mouse model, which demonstrated the significant efficacy of the MN-MXene-AS system in accelerating wound healing. These experimental results demonstrated the unique advantages of MNs in growth factor delivery and demonstrated the great potential of this system in promoting diabetic wound healing.^179^

As demonstrated in Fig. 8d, a comparative analysis of the microphenotypes and mechanical properties of the MN-PGA and MN-MXene-AS MN systems is provided. Scanning electron microscopy (SEM) images clearly revealed that, compared with MN-PGA, MN-MXene-AS has superior morphological characteristics, resulting in enhanced preservation postdemolding and increased mechanical strength. This design allows MN-MXene-AS to have greater advantages in penetrating the skin and performing efficient drug delivery, thus further promoting the effectiveness of diabetic wound healing.

Furthermore, Fig. 8e shows a novel porous MN array that highly mimics the microniche environment of stem cells. By encapsulating human adipose-derived stem cells (ADSCs) in Matrigel and loading them into porous MN arrays, a biomimetic growth environment is provided for ADSCs. The pore structure of the MN arrays provides ADSCs with sufficient space to proliferate and perform their promotional roles, and the MN arrays are mechanically strong enough to effectively penetrate the skin and deliver ADSCs to deeper regions of the wound. Taken together, these properties demonstrate the great potential of this microneedle system in diabetic wound healing, particularly in terms of tissue regeneration, collagen deposition and angiogenesis. Finally, Fig. 8f shows the growth of adipose stem cells on the porous microneedle array, with the results of fluorescence staining clearly showing that the ADSCs continued to proliferate on the surface of the microneedle arrays at 1, 3, 5 and 7 days after cell infusion, thus further validating the critical role of the porous microneedle arrays in supporting cell growth and promoting wound healing.^180^

In the following discussion, recent advances in the field of microneedles are explored, with a particular focus on composite innovations. The discussion will encompass the composite application of MNs with stem cells, nanomaterials, and growth factors. These innovative designs have been shown to increase drug delivery efficiency, thereby promoting the healing of diabetic wounds through the synergistic effect of bioactives. These innovations suggest that microneedle technology provides a more comprehensive and efficient solution for diabetic wound treatment, with broad clinical application prospects.

### 6.5 Combination approaches

**Fig. 9:**
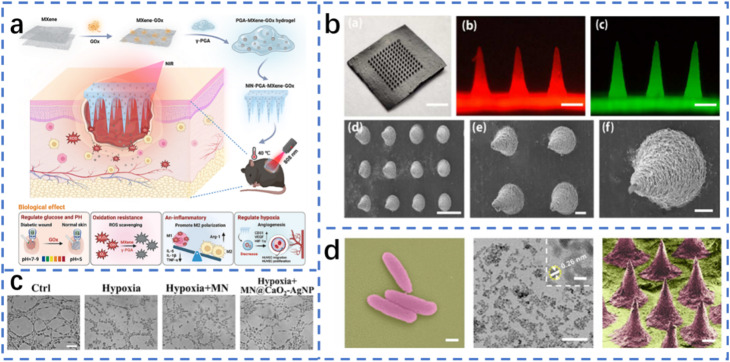
(a) Schematic illustration of the MXene-based MN patch (MN-PGA-MXene-GOx) and its mechanism for accelerating diabetic wound healing (created with BioRender).^181^ Reprinted with permission. Copyright 2024 Elsevier. (b) Optical image of the MN patch (scale bar, 2.5 mm); fluorescence microscopy images of RhB- and fluorescein-loaded MN tips (scale bars, 200 μm); SEM images of the MNs [scale bars, 500 μm (d), 100 μm (e), and 50 μm (f)].^182^ Reprinted with permission. Copyright 2023 AAAS. (c) HUVEC tube formation after 4 h of different treatments.^183^ Reprinted with permission. Copyright 2024 Elsevier. (d) SEM image of HMPs (purple: HMPs, scale bar = 200 nm); TEM image of ZnO (scale bar = 50 nm); SEM image of the MN patch (purple: MN tips; scale bar = 200 μm).^184^ Reprinted with permission. Copyright 2023 American Chemical Society.

The process of healing chronic diabetic wounds is influenced by a number of factors, including hyperglycemia, oxidative stress, an absence of oxygen, and microbial infections. Achieving the desired results with a single treatment is often challenging, and integrated multifunctional composite microneedle technology provides a more efficient solution for wound repair. In recent years, researchers have developed a series of innovative microneedles by combining materials engineering, nanotechnology, biocatalysis and physical stimulation to achieve integrated regulation of the diabetic wound microenvironment (Fig. 9).

By encapsulating Ti_3_C_2_ MXene nanosheets loaded with glucose oxidase (GOx), MN-PGA-MXene-GOx, which is based on poly(γ-glutamic acid) (γ-PGA), achieves the dual roles of smart glucose lowering and microenvironmental regulation. The latter catalyzes the oxidation of glucose to reduce the local glucose concentration while regulating the pH value to optimize the wound healing environment. The near-infrared (NIR) photothermal effect of MXenes enhances the catalytic activity of GOx under external irradiation, accelerating the process of glucose metabolism. In addition, the antioxidant properties of MXene and γ-PGA reduce the level of oxidative stress and decrease the degree of tissue damage caused by hydrogen peroxide (H_2_O_2_) produced during glucose oxidation. Studies have shown that MNs can effectively promote fibroblast proliferation, migration and neovascularization, thereby accelerating wound repair.

The self-powered enzyme-linked microneedle (MN) system forms a cascade of catalytic reactions with GOx and horseradish peroxidase (HRP), enabling it to reduce local hyperglycemia more efficiently than single enzyme-catalyzed microneedles. Furthermore, the system generates stable microcurrents that can further promote tissue repair. In this process, GOx oxidizes glucose to gluconic acid, while HRP catalyzes the resulting H_2_O_2_ to optimize the local microenvironment. Moreover, studies have shown that microcurrents promote fibroblast activation, enhance cell adhesion and migration, and accelerate angiogenesis. In addition, microcurrents can inhibit the growth of pathogens and reduce the risk of wound infection. Experimental data demonstrated that diabetic wounds treated with this self-powered microneedle exhibited accelerated and more complete healing, along with effective scar formation reduction, thereby providing further validation of the synergistic benefits of enzymatic glucose reduction and microelectrical stimulation.^181,182^

In the context of a diabetic wound, the hypoxic state imposes significant limitations on tissue repair capacity, underscoring the research priority of microneedles capable of active oxygenation. Antimicrobial oxygen-producing filipin protein-hydrogel MNs (MN@CaO_2_-AgNPs) combine calcium peroxide (CaO_2_) and silver nanoparticles (AgNPs) to provide antimicrobial effects, while the sustained release of oxygen effectively ameliorates hypoxic conditions. The slow hydrolysis of CaO_2_ releases oxygen in wound environments to improve fibroblast and vascular endothelial cell activity, which in turn accelerates tissue repair. AgNPs, on the other hand, have been shown to be effective at inhibiting the growth of various pathogens and reducing the risk of infection. In addition, MNs effectively promoted vascular neovascularisation and significantly enhanced the tube formation ability of HUVECs. Animal studies have shown that MNs can significantly increase the healing speed of diabetic wounds and reduce inflammatory responses.^183^

In addition, near-infrared (NIR)-responsive H-Z-MN-VEGF&bFGF MNs combined with graded particles (HMPs) and zinc oxide (ZnO) nanoparticles not only produce a photothermal effect to enhance antimicrobial ability under NIR irradiation but also promote vascular regeneration. Hair-derived HMPs have the ability to scavenge reactive oxygen species (ROS), thus preventing damage to blood vessels caused by ROS, while the addition of ZnO further enhances the antimicrobial activity of MNs. Moreover, the MNs were able to load vascular endothelial growth factor (VEGF) and basic fibroblast growth factor (bFGF), which together promoted vascular regeneration. The experiments revealed that the MNs effectively improved local blood flow in diabetic model animals and significantly enhanced collagen deposition and wound closure ability during wound repair.

As demonstrated by the preceding discussion, it is challenging for a single treatment to satisfy the numerous requirements of diabetic wound repair. Composite microneedle technology, however, has been shown to achieve comprehensive optimization of the diabetic wound microenvironment by virtue of its multifunctional synergistic effects. Enzyme-responsive MNs improve the local environment by lowering glucose and regulating pH. Self-powered MNs promote tissue repair *via* enzyme cascade catalysis and microelectrical stimulation, whereas oxygen-producing antimicrobial MNs alleviate hypoxia and provide antimicrobial protection. Finally, near-infrared photothermal microneedles integrate photothermal, antimicrobial, and angiogenic capabilities. Experimental studies have demonstrated that these innovative microneedles accelerate the healing process of diabetic wounds to different degrees and demonstrate good biocompatibility and application prospects. However, further in-depth studies on the long-term safety, histocompatibility and large-scale preparation process of MNs are still needed to ensure their effectiveness and feasibility in clinical practice.^184^ In conclusion, composite microneedle technology offers a novel and effective therapeutic strategy for the treatment of diabetic wounds and is anticipated to represent a significant breakthrough in the field of wound repair in the future.

## 7. Microneedle optimization for clinical translation

The clinical translation of microneedle (MN) platforms for diabetic wound therapy demands optimization that bridges laboratory innovation with the complex realities of patient care.^185–187^ While experimental systems have demonstrated impressive capabilities in glucose regulation, antimicrobial action, reactive oxygen species (ROS) scavenging, and angiogenesis promotion, many prototypes remain constrained by limitations in mechanical robustness, penetration consistency, long-term stability, and large-scale manufacturability. Clinical-grade MNs must not only deliver precise therapeutic doses but also perform reliably across diverse patient populations with variable skin characteristics and wound conditions. For diabetic wounds in particular, the device must sustain bioactivity in harsh, infection-prone environments while remaining minimally invasive and comfortable for repeated application. These requirements necessitate a holistic optimization approach that simultaneously addresses therapeutic performance, safety, usability, and compliance with regulatory and manufacturing standards.^188^

Optimization strategies for bio-inspired polymeric MNs focus on harmonizing materials chemistry, structural design, and functional integration. Biodegradable polymers such as hyaluronic acid, polylactic acid, and silk fibroin can be engineered with tunable mechanical strength and dissolution rates to ensure both skin penetration and controlled release. Incorporating porous metal–organic frameworks (MOFs) loaded with glucose oxidase allows dynamic glucose depletion and *in situ* generation of hydrogen peroxide for antimicrobial action, while co-loading angiogenic peptides or growth factors accelerates tissue repair. Structural refinements enhance penetration efficiency and reduce tissue trauma. Formulation strategies, including nanoparticle encapsulation and lyophilization, improve the stability of sensitive bioactive agents during storage and transport. Advances in micro-molding, lithography, and additive manufacturing now enable precise, reproducible fabrication of complex MN architectures, paving the way for consistent large-scale production.^189–192^

Nevertheless, key challenges must be resolved to achieve broad clinical adoption. Regulatory approval processes for multifunctional MN systems that combine drug delivery, sensing, and wound monitoring remain complex, particularly when multiple bioactive agents are involved. Long-term biocompatibility and safety data under repeated application in chronic wound settings are still limited. Manufacturing at scale must ensure consistent drug loading, release kinetics, and structural integrity, all while maintaining cost-effectiveness to facilitate adoption in diverse healthcare settings. Future optimization will likely involve integrating real-time biochemical sensing with responsive release systems, enabling MNs to adapt therapy to wound status dynamically. Standardized preclinical and clinical evaluation protocols will be critical to accelerating regulatory review and fostering clinician confidence. By strategically advancing materials design, structural engineering, and manufacturing readiness, bio-inspired polymeric MN platforms can evolve from promising experimental tools into clinically viable, patient-centered solutions for the effective treatment of diabetic wounds.

## 8. Conclusions and perspectives

Microneedle (MN)-based therapeutic platforms have emerged as a transformative advancement in diabetic wound management, providing a minimally invasive, patient-friendly, and multifunctional alternative to traditional treatments. Chronic diabetic wounds present unique therapeutic challenges due to their multifactorial pathology, which includes persistent hyperglycemia, bacterial colonization, oxidative stress, and impaired angiogenesis. Conventional strategies such as systemic antibiotics, topical agents, and growth factor dressings often fail to address all these factors simultaneously, leading to suboptimal healing and high recurrence rates. In this context, MN systems have demonstrated remarkable potential by enabling precise, localized delivery of therapeutic agents directly into the wound bed, thereby overcoming barriers to drug penetration and reducing systemic exposure. Their ability to integrate multiple therapeutic mechanisms into a single, easy-to-use platform positions them at the forefront of next-generation wound care technologies.

The recent convergence of materials science, bioengineering, and wound pathology has expanded the functional capabilities of MN platforms far beyond simple transdermal delivery. Bioinspired structural designs improve skin penetration efficiency while minimizing tissue trauma, and advanced biodegradable polymers enable controlled drug release and biocompatibility. Incorporating porous metal–organic frameworks loaded with glucose oxidase allows MNs to dynamically regulate glucose levels while generating antimicrobial hydrogen peroxide, effectively addressing both metabolic dysregulation and infection. Additional integration of angiogenic peptides, anti-inflammatory agents, or oxygen-generating components further enhances tissue regeneration. These multifunctional designs not only address the complex microenvironment of diabetic wounds but also offer the potential for reduced treatment frequency, improved patient comfort, and higher adherence to therapy. The cumulative effect of these innovations is a wound care modality that is both mechanistically comprehensive and highly adaptable to different stages of healing.

Looking ahead, the next phase of MN optimization will depend on a coordinated approach that combines material innovation, structural refinement, and functional adaptability. Hybrid composites incorporating natural polymers, bioactive nanoparticles, and responsive hydrogels can enhance mechanical robustness, biodegradability, and therapeutic responsiveness. Structural advancements such as gradient stiffness profiles, hierarchical porosity, and microfluidic-assisted architectures can improve fluid drainage, optimize drug penetration depth, and enable multi-phase release tailored to wound healing dynamics. The integration of biosensing elements capable of detecting pH changes, infection biomarkers, glucose concentration, or oxygen saturation will enable MN systems to evolve into intelligent, closed-loop platforms. These smart MNs could automatically adjust drug release in response to real-time wound conditions, merging diagnosis and therapy into a single device and opening new possibilities for personalized wound management.

Despite these promising developments, several challenges must be addressed before MN systems can achieve widespread clinical adoption. Scalable, reproducible, and cost-effective manufacturing processes must be optimized to deliver consistent product quality and performance. Regulatory approval for multifunctional MNs, especially those delivering biologics or combining therapeutic and diagnostic capabilities, requires rigorous demonstration of safety, sterility, and stability. Long-term clinical validation through large-scale, randomized trials will be essential to establish comparative efficacy, durability of healing, recurrence rates, and cost-effectiveness relative to standard-of-care therapies. Furthermore, patient-centered design considerations will play a pivotal role in adoption. With sustained interdisciplinary collaboration among materials scientists, engineers, clinicians, and regulatory experts, MN platforms have the potential to transition from promising experimental prototypes into integral components of precision, adaptive, and accessible diabetic wound care, ultimately improving patient outcomes and reducing the global burden of chronic wounds.

## Conflicts of interest

The authors declare that they have no conflicts of interest.

## Data Availability

No primary research results, software or code have been included and no new data were generated or analysed as part of this review.
